# An experimental comparison of different hierarchical self-tuning regulatory control procedures for under-actuated mechatronic systems

**DOI:** 10.1371/journal.pone.0256750

**Published:** 2021-08-30

**Authors:** Omer Saleem, Khalid Mahmood-ul-Hasan, Mohsin Rizwan

**Affiliations:** 1 Department of Electrical Engineering, National University of Computer and Emerging Sciences, Lahore, Pakistan; 2 Department of Electrical Engineering, University of Engineering and Technology, Lahore, Pakistan; 3 Department of Mechatronics and Control Engineering, University of Engineering and Technology, Lahore, Pakistan; Nankai University, CHINA

## Abstract

This paper presents an experimental comparison of four different hierarchical self-tuning regulatory control procedures in enhancing the robustness of the under-actuated systems against bounded exogenous disturbances. The proposed hierarchical control procedure augments the ubiquitous Linear-Quadratic-Regulator (LQR) with an online reconfiguration block that acts as a superior regulator to dynamically adjust the critical weighting-factors of LQR’s quadratic-performance-index (QPI). The Algebraic-Riccati-Equation (ARE) uses these updated weighting-factors to re-compute the optimal control problem, after every sampling interval, to deliver time-varying state-feedback gains. This article experimentally compares four state-of-the-art rule-based online adaptation mechanisms that dynamically restructure the constituent blocks of the ARE. The proposed hierarchical control procedures are synthesized by self-adjusting the (i) controller’s degree-of-stability, (ii) the control-weighting-factor of QPI, (iii) the state-weighting-factors of QPI as a function of “state-error-phases”, and (iv) the state-weighting-factors of QPI as a function of “state-error-magnitudes”. Each adaptation mechanism is formulated via pre-calibrated hyperbolic scaling functions that are driven by state-error-variations. The implications of each mechanism on the controller’s behaviour are analyzed in real-time by conducting credible hardware-in-the-loop experiments on the QNET Rotary-Pendulum setup. The rotary pendulum is chosen as the benchmark platform owing to its under-actuated configuration and kinematic instability. The experimental outcomes indicate that the latter self-adaptive controller demonstrates superior adaptability and disturbances-rejection capability throughout the operating regime.

## 1. Introduction

The design principles of under-actuated self-stabilizing systems are extensively used in the fabrication of humanoid robotic systems, aeronautical systems, self-balancing transporters, robotic manipulators, and underwater vehicles, etc [1, 2]. These systems have high dexterity, better control-input economy, and a lesser propensity to break down [3]. However, the under-actuated systems have fewer actuators than the degrees-of-freedom to be regulated [4]. The system’s under-actuated configuration, nonlinear dynamics, and open-loop instability poses a challenging problem to the researchers in developing robust controllers that can effectively reject the exogenous disturbances encountered by the physical system in real-time applications [5, 6].

### 1.1. Literature review

Conventional controllers have been extensively used to optimize the disturbance compensation behavior of the aforementioned class of mechatronic systems [7, 8]. The integer-order PID controllers are widely preferred in the control industry due to their simple structure and reliable control effort [9]. However, they cannot efficiently mitigate the influence of parametric uncertainties owing to their limited degrees-of-freedom and simple structure [10]. The fractional-order PID controllers offer relatively better flexibility of controller design, which increases the controller’s degrees-of-freedom and enables it to quickly reject the nonlinear disturbances [11]. However, tuning the controller parameters is an ill-posed problem [12]. Despite their enhanced flexibility, the fuzzy controllers require a large number of empirically-defined qualitative rules to deliver robust control decisions [13]. Apart from degrading the controller’s computational economy, this arrangement also increases the human-rendered inaccuracies in the synthesized rule-base [14]. The neural controllers require rigorous training and large sets of training-data to deliver an accurate data-driven control model [15]. They also puts an excessive recursive computational burden on the digital computer [16]. The Linear-Quadratic-Regulator (LQR) is a state-space control procedure that minimizes a quadratic-performance-index (QPI), which captures the state and control-input variations, to compute an optimal set of state-feedback gains [17, 18]. Despite its optimality and guaranteed stability, the LQR lacks robustness against exogenous disturbances, model variations, and identification errors [19, 20]. The robustness of the generic LQR can be improved by prescribing a “Degree-of-Stability” (DoS) in its structure [21]. The DoS design relocates the system’s eigenvalues on the left-hand of the line *s* = −*β* in the complex plane, where, "*s*" is the Laplace operator and *β*>0 is a preset parameter that defines the LQR’s DoS [22]. The repositioning of eigenvalues enhances the controller’s response speed and its damping against exogenous disturbances by manipulating its phase-margin [23]. However, this technique compromises the control-input expenditure of the controller [24].

The robust nonlinear controllers put unnecessary restraints on deriving the exact solution due to the boundary conditions and complex geometry of the system’s model [5, 25]. The nonlinear control scheme proposed in [26] effectively handles the actuated state-constraints, un-actuated state-constraints, and composite variable constraints for a specific class of under-actuated systems. However, it does not address the effect of parametric uncertainties encountered by the system. The sliding-mode controllers are also renowned for delivering robust control efforts [27]. However, they apply highly discontinuous control force which inevitably injects chattering in the response [28]. The back-stepping controllers are also used to regulate the performance of the nonlinear systems [29]. However, the cancellation of indefinite cross-coupling terms, which is done to maintain the negativity of the Lyapunov function’s first-derivative throughout the operating regime, contributes to a higher control activity and degrades the system’s robustness as well [30].

The adaptive controllers are used as an important tool in disturbance-compensators for under-actuated systems [31]. They perform on-board reasoning to dynamically restructure the control procedure by self-tuning the critical controller parameters [32]. This setup enables the system to quickly adapt to the abrupt state-variations [32–34]. Historically, the adaptive controllers are categorized via their direct or indirect nature. The direct approach self-adjusts the critical controller-parameters as a function of the error-variables [13]. In the indirect approach, an identification scheme is used to estimate the system’s unknown model-parameters to update the control law [14].

Extensive research has been done to synthesize robust adaptive controllers for under-actuated mechatronic systems [35, 36]. The Model-Reference-Adaptive-Controllers utilizes the Lyapunov theory to track a reference control model which leads to the online dynamic adjustment of the critical controller parameters [37, 38]. However, identifying an accurate reference model for the tracking purpose is a difficult task [39]. The gain-scheduling mechanism employs a state-driven look-up table to select pre-configured feedback controllers; where, each controller is designed specifically for a given operating condition [40]. The calibration and stability assurance of the constituent controllers for a system with a big range of uncertainty become quite laborious [41]. The model-predictive-controllers use smaller time frames to solve the receding-horizon optimization problem and deliver time-varying controller gains [42]. However, they render wrong predictions which may lead to a fragile control effort under long drifting disturbances or model variations [43]. The State-Dependent-Riccati-Equation based controllers require accurate state-dependent-coefficient matrices to update the Riccati Equation solutions [44]. However, an accurate definition of these matrices is quite hard due to the restrictions imposed by the nonlinear dynamics of higher-order systems [45]. The Markov-Jump-Linear-System is a stochastic control technique that is renowned for its reliance against the random faults occurring in the cyber-physical system [46]. However, the cost and likelihood of acquiring accurate a priori transition probabilities for necessary computations is expensive and arguable, respectively [47].

The state-error-driven nonlinear scaling functions have also been extensively used for the development of expert adaptive systems to online adapt the controller parameters [48]. Retrofitting the linear compensators with nonlinear scaling functions to adaptively modify the critical controller gains has garnered a lot of traction in developing robust control for non-minimum phase systems [49]. The nonlinear-type feedback controllers tend to improve the system’s damping against oscillations, reference-tracking accuracy, and error-convergence rate [50, 51]. There are two main categories of nonlinear-type gain adaptation laws that are widely used in the adaptive control field; namely, the state-error-magnitude observers and state-error-phase observers. In state-error-magnitude observers, the online dynamic gain-adjustment depends on the magnitude of the state-error variable and its higher-order derivatives [52]. In state-error-phase observers, the online dynamic gain-adjustment is driven by the magnitude of the classical state-error as well as the direction of motion of the state response (commonly referred to as the “phase” of the state-response) [53]. The phase information helps in flexibly manipulating the controller’s characteristics as the response deviates from or converges to the reference [54]. The biologically-inspired artificial-immune system is a computationally intelligent adaptive mechanism that efficiently rejects the exogenous disturbances [55]. They mimic the self-regulation capability of biological immune systems to adaptively tune the controller parameters which optimizes the controller’s adaptability to environmental indeterminacies [56].

The hierarchical self-tuning state-feedback regulators are yet another emerging control paradigm [57, 58]. They are implemented by dynamically adjusting the constituent weighting matrices of LQR’s QPI to indirectly modify the controller gains [59]. The online variations in these weighting-factors manipulates the critical parameters in the succeeding layers of the controller’s structure which eventually delivers time-varying state-feedback gains.

### 1.2. Proposed approach

The main contribution of this article is the development and experimental comparison of four unique state-of-the-art nonlinear-type hierarchical self-tuning state-feedback regulators for under-actuated mechatronic systems in order to improve their disturbance-rejection capability against exogenous disturbances. The proposed control scheme follows a hierarchical architecture that re-computes the state-feedback gains, after every sampling-interval, based on the state-error-dependent adaptive tuning of weighting-factors associated with LQR’s quadratic-performance-index (QPI). For this purpose, the generic LQR structure is retrofitted with an auxiliary online self-tuning mechanism that acts as a superior regulator to adaptively tune the constituent weighting-factors associated with the QPI. The Riccati equation uses these adjusted weights to deliver the time-varying state-feedback gains. Each self-tuning mechanism is designed such that it exploits a specific aspect of the system’s state-error profile and harnesses it to effectively reposition the system’s closed-loop eigenvalues in the stable region of the complex-plane. The said hierarchical control procedure is quite innovative because, apart from adjusting the state-feedback gains online, the solution of Riccati equation concurrently guarantees the asymptotic-convergence of the control law as long as the concerned weighting-factors are varied within pre-defined bounds. Hence, additional stability proofs are not required. The salient innovative contributions of this article are postulated as follows:

1. Development of a self-tuning mechanism for the LQR’s “degree-of-stability”.
2. Development of a self-tuning mechanism for the control-weighting-factor associated with the QPI.
3. Development of a self-tuning mechanism for the state-weighting-factors of QPI that depends on the system’s state-error-phase.
4. Development of a self-tuning mechanism for the state-weighting-factors of QPI that depends on the magnitudes of the system’s state-error variables.
5. Formulation of each self-tuning mechanism by using pre-configured hyperbolic functions to re-scale the critical weighting-factors in real-time.
6. Comparative performance assessment of the proposed self-tuning controller variants by conducting credible real-time experiments, designed specifically to emulate practical disturbance scenarios in the physical environment, on the standard QNET Rotary Pendulum setup [11].

The experimental results (shown later in this article) indicate that each self-tuning-regulator variant significantly enhances the system’s robustness against the exogenous disturbances and the control-input economy to a certain degree while preserving the system’s asymptotic-stability throughout the operating regime. The experimental comparison of the four different structures of hierarchical self-tuning regulators, that employ innovative rule-based adaptation mechanisms to dynamically adjust the critical weighting-factors of the QPI, has not been attempted previously in the open literature. Hence, this is the main focus of this article.

The remaining paper is organized as follows: The pendulum system is mathematically modeled in Section 2. The baseline fixed-gain LQR is synthesized in Section 3. The detailed design of the four prescribed hierarchical self-tuning regulators is presented in Section 4. The experimental comparison of the proposed self-tuning regulator is presented in Section 5. The paper is concluded in Section 7.

## 2. System model

**Fig 1:**
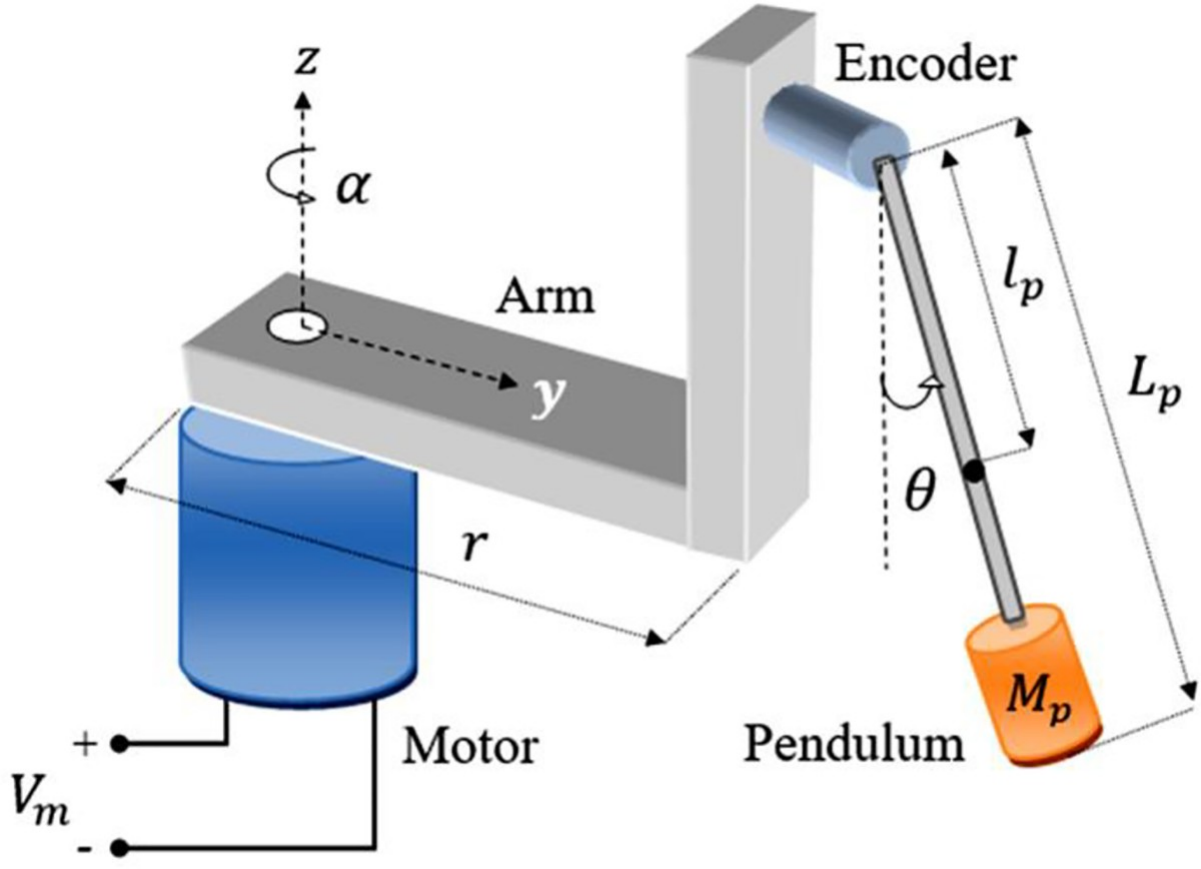
Hardware schematic of a typical rotary pendulum system.

In this paper, a standard rotary inverted pendulum (RIP) system is used as the benchmark platform to experimentally analyze the implications of the proposed control procedure [60]. It requires an active control system to stabilize itself vertically. Apart from being under-actuated in nature, the said multivariable system also exhibits all the properties typically associated with mechatronic systems; such as open-loop (or kinematic) instability, complex geometry, and nonlinear dynamics [61]. The block diagram of an RIP system is illustrated in Fig 1. The system employs a DC geared servo-motor to apply the necessary control torque to rotate the pendulum’s arm, which is coupled to the motor’s shaft. The arm’s angular displacement energizes the pendulum rod to swing-up and balance itself vertically. The angular-displacements of the arm and the rod are denoted as *α* and *θ*, respectively.

The system’s nonlinear equations of motion are formulated via the Euler-Lagrange approach [62]. The system’s Lagrangian (*L*), expressed in Eq 1, is evaluated by computing the difference between the total kinetic energy (*T*) and the total potential energy (*V*) of the system is computed in terms of the coordinates (*φ* and *θ*) and their corresponding angular-velocities ($\dot{\varphi }$ and $\dot{\theta }$).


L=T−V (1)

The Euler-Lagrange equations of the RIP system are derived as follows [62].

$$\frac{\delta }{\delta t}\left(\frac{\delta L}{\delta \dot{\alpha }}\right)-\frac{\delta L}{\delta \alpha }=\tau ,\frac{\delta }{\delta t}\left(\frac{\delta L}{\delta \dot{\theta }}\right)-\frac{\delta L}{\delta \theta }=0$$ (2)

where, *τ* represents the torque applied by the DC motor. It is expressed as follows.


$$\tau =\frac{K_{t}(V_{m}-K_{m}\dot{\alpha })}{R_{m}}$$ (3)

The viscous damping forces and frictional forces are neglected in this research. The resulting nonlinear relationship between *α*, *θ*, and *τ* is expressed as follows [62].


$$\ddot{\alpha }=\frac{-rM_{p}^{2}l_{p}^{2}g(\cos\;\theta )\theta -J_{p}M_{p}r^{2}\;\cos\;\theta \;\sin\;\theta {\left(\dot{\alpha }\right)}^{2}-(J_{p}+M_{p}l_{p}^{2})\tau }{(M_{p}r^{2}(\sin^{2}\theta )-J_{e}-M_{p}r^{2})J_{p}-M_{p}l_{p}^{2}J_{e}},$$

$$\ddot{\theta }=\frac{-M_{p}l_{p}(\left(M_{p}r^{2}g\;\sin^{2}\;\theta -J_{e}g-M_{p}r^{2}g)\theta +r(J_{e}{\dot{\alpha }}^{2}\;\sin\;\theta -\tau \;\cos\;\theta \right))}{(M_{p}r^{2}(\sin^{2}\theta )-J_{e}-M_{p}r^{2})J_{p}-M_{p}l_{p}^{2}J_{e}}.$$ (4)

The aforementioned set of nonlinear equations can be linearized around the point $\alpha =\theta =\dot{\alpha }=\dot{\theta }=0$. Furthermore, the small-angular displacements of the pendulum rod are approximated via the following expressions.


sinθ≈θ,cosθ≈1 (5)

The state-space model of a linear dynamical system is represented via Eq 6 [11].

$$\dot{x}=Ax+Bu,y=Cx+Du$$ (6)

where, *x* is the state-vector, *y* is the output-vector, *u* is the control input signal, *A* is the state-transition matrix, *B* is the input matrix, *C* is the output matrix, and *D* is the feed-forward matrix. The state-vector and the control input-vector of the RIP system are identified in Eq 7 [59].

$$x={\left[\alpha \;\theta \;\dot{\alpha }\;\dot{\theta }\right]}^{T},u=V_{m}$$ (7)

where, *V*_*m*_ is the control-input voltage applied to operate the DC motor. The nominal state-space model of the RIP system is presented as follows [59].

$$A=[\begin{array}{cccc} 0 & 0 & 1 & 0\\ 0 & 0 & 0 & 1\\ 0 & a_{1} & a_{2} & 0\\ 0 & a_{3} & a_{4} & 0 \end{array}],B=[\begin{array}{c} 0\\ 0\\ b_{1}\\ b_{2} \end{array}],C=[\begin{array}{cccc} 1 & 0 & 0 & 0\\ 0 & 1 & 0 & 0\\ 0 & 0 & 1 & 0\\ 0 & 0 & 0 & 1 \end{array}],D=[\begin{array}{c} 0\\ 0\\ 0\\ 0 \end{array}].$$ (8)

where,

$$a_{1}=\frac{rM_{p}^{2}l_{p}^{2}g}{J_{p}J_{e}+J_{e}l_{p}^{2}M_{p}+J_{p}M_{p}r^{2}},a_{2}=\frac{-K_{t}K_{m}(J_{p}+M_{p}l_{p}^{2})}{(J_{p}J_{e}+J_{e}l_{p}^{2}M_{p}+J_{p}M_{p}r^{2})R_{m}},$$

$$a_{3}=\frac{M_{p}l_{p}g(J_{e}+M_{p}r^{2})}{J_{p}J_{e}+J_{e}l_{p}^{2}M_{p}+J_{p}M_{p}r^{2}},a_{4}=\frac{-rM_{p}l_{p}K_{t}K_{m}}{(J_{p}J_{e}+J_{e}l_{p}^{2}M_{p}+J_{p}M_{p}r^{2})R_{m}},$$

$$b_{1}=\frac{K_{t}(J_{p}+M_{p}l_{p}^{2})}{(J_{p}J_{e}+J_{e}l_{p}^{2}M_{p}+J_{p}M_{p}r^{2})R_{m}},b_{2}=\frac{rM_{p}l_{p}K_{t}}{(J_{p}J_{e}+J_{e}l_{p}^{2}M_{p}+J_{p}M_{p}r^{2})R_{m}}$$

The model parameters of the QNET RIP are identified in Table 1 [11].

**Table 1:** Model parameters of QNET Rotary Pendulum.

Parameter	Description	Identified value
*M*_*p*_	Mass of pendulum	0.027 kg
*l*_*p*_	Pendulum center of mass	0.153 m
*L*_*p*_	Length of pendulum rod	0.191 m
*r*	Length of horizontal arm	0.083 m
*M*_*arm*_	Mass of arm	0.028 kg
*g*	Gravitational acceleration	9.810 m/s^2^
*J*_*e*_	Moment about motor shaft	1.23×10^−4^ kgm^2^
*J*_*p*_	Moment about pendulum	1.10×10^−4^ kgm^2^
*R*_*m*_	Motor armature resistance	3.30 Ω
*L*_*m*_	Motor armature inductance	47.0 mH
*K*_*t*_	Motor torque constant	0.028 N.m
*K*_*m*_	Back e.m.f. constant	0.028 V/(rad/s)
*T*_*m*_	Maximum torque	0.14 Nm

## 3. Linear quadratic regulator

The LQR is a standard state-space control strategy that is widely favored for optimal position-regulation of multivariable electro-mechanical systems [19]. The LQR yields an optimal control trajectory by minimizing an energy-like QPI, expressed in Eq 9, that captures the state-variations and the control input associated with the linear dynamical system [17].

$$J_{lq}=\frac{1}{2}\int_{0}^{\infty }\left[{x(t)}^{T}Qx(t)+{u(t)}^{T}Ru(t)\right]dt$$ (9)

where, ***Q*** ∈ ℝ^4×4^ and ***R*** ∈ ℝ are the state and control-input weighting matrices, respectively. The QPI minimization is followed by the solution of Hamilton-Jacobi-Bellman (HJB) equation to acquire the state-feedback gains offline [17]. The weighting-matrices are selected such that ***Q*** is a positive semi-definite matrix and ***R*** is a positive definite matrix. For the RIP system considered in this research, the ***Q*** and ***R*** matrices are symbolically represented as shown in Eq 10.

$$Q=diag(q_{\varphi }\;q_{\theta }\;q_{\dot{\alpha }}\;q_{\dot{\theta }}),\;R=\rho$$ (10)

where, *q*_*x*_ and *ρ* represent the real-numbered coefficients of the ***Q*** and ***R*** matrices, respectively. The value of *ρ* is selected as unity to maintain a reasonable control-input economy. The ***Q*** matrix is tuned in this research by iteratively minimizing the performance criterion given in Eq 11 to minimize the position-regulation error as well as the control-input energy [63].

$$J_{c}=\int_{0}^{\infty }{\left|e_{\alpha }(t)\right|}^{2}+{\left|e_{\theta }(t)\right|}^{2}+{\left|u(t)\right|}^{2}dt$$ (11)

$such\;that,\;e_{\alpha }(t)=\alpha_{ref}-\alpha (t),e_{\theta }(t)=\pi -\theta (t)$ where, *e*_*α*_(*t*) and *e*_*θ*_(*t*) represent the error in the angular displacement of arm and rod from their corresponding reference positions, respectively. The reference position of the pendulum’s rod is set as *π* radians in order to stabilize it vertically. The angular position of the pendulum’s arm recorded at the beginning of every experimental trial is considered as its reference, *α*_*ref*_. The LQR delivers the optimal set of state-feedback gains with the lowest cost of *J*_*lq*_. However, these optimal gains are computed by using a specific set of ***Q*** and ***R*** matrices. This arrangement may not always contribute a good position-regulation behavior with respect to *J*_*lq*_. Hence, to optimize the selection procedure, *J*_*c*_ is used to tune the state-weighting factors in this research [59]. To acquire the best-fit solution, each state weighting-factor is selected from the range [0, 500] in this research. The search is initiated from a random point in the range-space. The search is conducted in the direction of descending gradient of *J*_*c*_ and it is terminated when the minimum cost is achieved. The coefficients of ***Q*** and ***R*** matrices acquire for this research (corresponding to the minimum cost of *J*_*c*_) are presented as follows.


Q=diag(32.852.26.12.5),R=1 (12)

The Algebraic-Riccati-Equation (ARE) utilizes the system’s nominal model as well as the tuned ***Q*** and ***R*** matrices to compute the solution, ***P***, as shown in Eq 13.

$$A^{T}P+PA-PBR^{-1}B^{T}P+Q=0$$ (13)

where, ***P***∈ℝ^*4×4*^, is a symmetric positive definite matrix. It is well-known that if the system is controllable and that ***Q*** = ***Q***^*T*^ ≥ 0 and ***R*** = ***R***^*T*^ ˃ 0, the solution of ARE yields an asymptotically-stable control behavior [17]. The state-feedback gain vector, ***K***_***f***_, is calculated as shown in Eq 14.

$$K=R^{-1}B^{T}P$$ (14)

where, $K=[k_{\alpha }\;k_{\theta }\;k_{\dot{\alpha }}\;k_{\dot{\theta }}]$. The optimal control law is expressed as follows.


$$u_{f}(t)=-Kx(t)$$ (15)

The evaluation of the gain vector yields K=[−6.21130.56−4.2217.83]. The linear control law is restructured by equipping it with the following state-error-integral variables.


$$\varepsilon_{\varphi }=\int_{0}^{t}e_{\varphi }(\tau )d\tau ,\varepsilon_{\theta }=\int_{0}^{t}e_{\theta }(\tau )d\tau$$ (16)

This augmentation improves the pendulum’s damping against fluctuations and its reference-tracking behavior [18]. The integral control law is expressed as follows.


$$u_{i}(t)=K_{i}\varepsilon (t)=[K_{i\varphi }\;K_{i\theta }]\left[\begin{array}{c} \varepsilon_{\varphi }\\ \varepsilon_{\theta } \end{array}\right]$$ (17)

The integral-gain vector ***K***_*i*_ is tuned by iteratively minimizing the cost function, *J*_*c*_, to minimize the position-regulation error. The ***K***_*i*_ vector that yields the minimum cost in the range [-5, 0] is selected. In this paper, the integral gains are tuned as $K_{i}=[-2.06\;-7.47\times 10^{-6}]$. The baseline control law is given by the linear combination of the optimal control law and the integral control law as shown in Eq 18.


$$u(t)=-K_{f}x(t)+K_{i}\varepsilon (t)$$ (18)

## 4. Hierarchical self-tuning-regulator design

The ubiquitous LQR uses the system’s linear state-space model to deliver fixed state-feedback gains. Thus, it lacks robustness against the state-deviations caused by the bounded disturbances, modeling uncertainties, identification errors, and other parametric variations. To solve this problem, the LQR is augmented with an online adaptation law that dynamically reconfigures the critical controller parameters. The adaptation law is realized by using state-error-dependent nonlinear scaling functions. These synthetic “nonlinear” functions flexibly manipulate the control profile to reject the exogenous disturbances. This arrangement significantly improves the controller’s adaptability and disturbance-rejection capability; although, the resulting self-tuning regulator continues to utilize the system’s linear state-space model.

This section presents the theoretical background and formulation of four different state-of-the-art hierarchical adaptive state-feedback control procedures. Each self-tuning mechanism adaptively modulates the gains of the LQR. The proposed mechanisms redesign the nominal LQR, after every sampling interval, to flexibly manipulate the control-input trajectory which aids in efficiently rejecting the exogenous disturbances and parametric variations. It is to be noted that only the state-feedback gains are being updated online in the proposed adaptive control procedures; whereas, the integral gains are kept fixed at $K_{i}=[-2.06\;-7.47\times 10^{-6}]$ as discussed in the previous section. Each proposed adaptive control procedure undertakes to achieve a beneficial compromise between the position-regulation behaviour and control energy expenditure while maintaining the system’s stability across a broad range of operating conditions. As discussed earlier, the proposed adaptation laws self-adjust specific parameters (existing naturally) within the hierarchical structure of the LQR control system. The online reconfiguration of these targeted parameter indirectly leads to the re-computation of state-feedback gains, after every sampling interval. In this article, four unique hierarchical self-tuning control procedures are investigated. These control procedures are individually synthesized by:

1. Self-adjusting the degree-of-stability of the LQR by using state-error feedback.
2. Self-adjusting the ***R*** matrix by using state-error feedback.
3. Self-adjusting the coefficients of ***Q*** matrixby using a well-established rationale that depends on the state-error-phase feedback.
4. Self-adjusting the ***Q*** and ***R*** matrices by using well-postulated meta-rules that depend on the state-error-magnitude feedback.

The adaptation laws are formulated via pre-calibrated hyperbolic nonlinear scaling functions. These functions are continuous which allows for a smooth variation of the concerned weights as the operating conditions change. These functions are bounded which limits the variation of the concerning weights and thus, ensures an asymptotically-stable control behaviour. The symmetry of the hyperbolic functions, about the vertical axis, helps to appropriately steer the control trajectory as the polarities of the state-error variables change. Finally, these algebraic equations can be easily solved in a single-step after every sampling interval. Unlike the iterative auto-tuning or gradient-descent techniques, the real-time computation of hyperbolic scaling functions does not put an excessive recursive computational burden on the embedded processor. Hence, they are computationally economical and can be easily programmed in the control software by using modern-day digital computers.

### 4.1. Adjustable degree-of-stability

The baseline LQR is transformed into a self-tuning-regulator by retrofitting it with a self-adjusting degree-of-stability (DoS) [21]. The QPI is equipped with a reconfiguration block that relocates the system’s closed-loop poles on the left-hand side of the vertical line, *s* = −*β*(*t*), on the complex *s* -plane; where, *β*(.) is a state-error dependent time-varying positive constant. The original QPI is modified by associating a time-varying exponential multiplying factor of the form *e*^2*β*(*t*)*t*^ with it as shown in Eq 19 [22].


$$J_{lq}^{*}=\frac{1}{2}\int_{0}^{\infty }e^{2\beta (t)t}[{x(t)}^{T}Qx(t)+{u(t)}^{T}Ru(t)]dt$$ (19)

The multiplication of the typical cost-function with the time-varying exponential term shifts the position of eigenvalues of the state-transition matrix ***A*** on the left side of the line *s* = −*β*(*t*) which ensures the asymptotic-stability of the controller’s operation [22]. The revised cost-function can be simplified according to the following expression.


$$J_{lq}^{*}=\frac{1}{2}\int_{0}^{\infty }\left[{\left(e^{\beta (t)t}x(t)\right)}^{T}Q(e^{\beta (t)t}x(t))+{\left(e^{\beta (t)t}u(t)\right)}^{T}R(e^{\beta (t)t}u(t))\right]dt$$ (20)

This simplification implies that the expressions of the state-vector, as well as the control-input vector, can be revised as expressed below [23].


$$\bar{x}(t)=e^{\beta (t)t}x(t),\bar{u}(t)=e^{\beta (t)t}u(t)$$ (21)

The substitution of the revised expressions of the state-vector and control-input vector yields the following expression of the cost-function.


$$J_{lq}^{*}=\frac{1}{2}\int_{0}^{\infty }\left[{\bar{x}(t)}^{T}Q\bar{x}(t)+{\bar{u}(t)}^{T}R\bar{u}(t)\right]dt$$ (22)

The system’s state-equation is also modified as expressed below [40].


$$\dot{\bar{x}(t)}=(A+\beta (t)I)\bar{x}(t)+B\bar{u}(t)$$ (23)

The expression in the Eq (18) reveals that the augmentation of the exponential term, *e*^2*β*(*t*)*t*^, in the quadratic cost-function ends up transforming the system’s state-matrix ***A*** into ***A***+*β*(*t*)***I***. Hence, this arrangement contributes in varying the coefficients of the state-matrix as a function of the state-variables. The modified expression of ARE is shown below [24].


$${\left(A+\beta (t)I\right)}^{T}P(t)+P(t)(A+\beta (t)I)-P(t)BR^{-1}B^{T}P(t)+Q=0$$ (24)

The time-varying state-feedback gain vector is updated online as follows.


$$K_{d}(t)=R^{-1}B^{T}P(t)$$ (25)

The updated gain vector, ***K***_***d***_(*t*), flexibly steers the control trajectory using the following Self-Tuning-Regulator (STR) control law.


$$u(t)=-K_{d}(t)x(t)+K_{i}\varepsilon (t)$$ (26)

**Fig 2:**
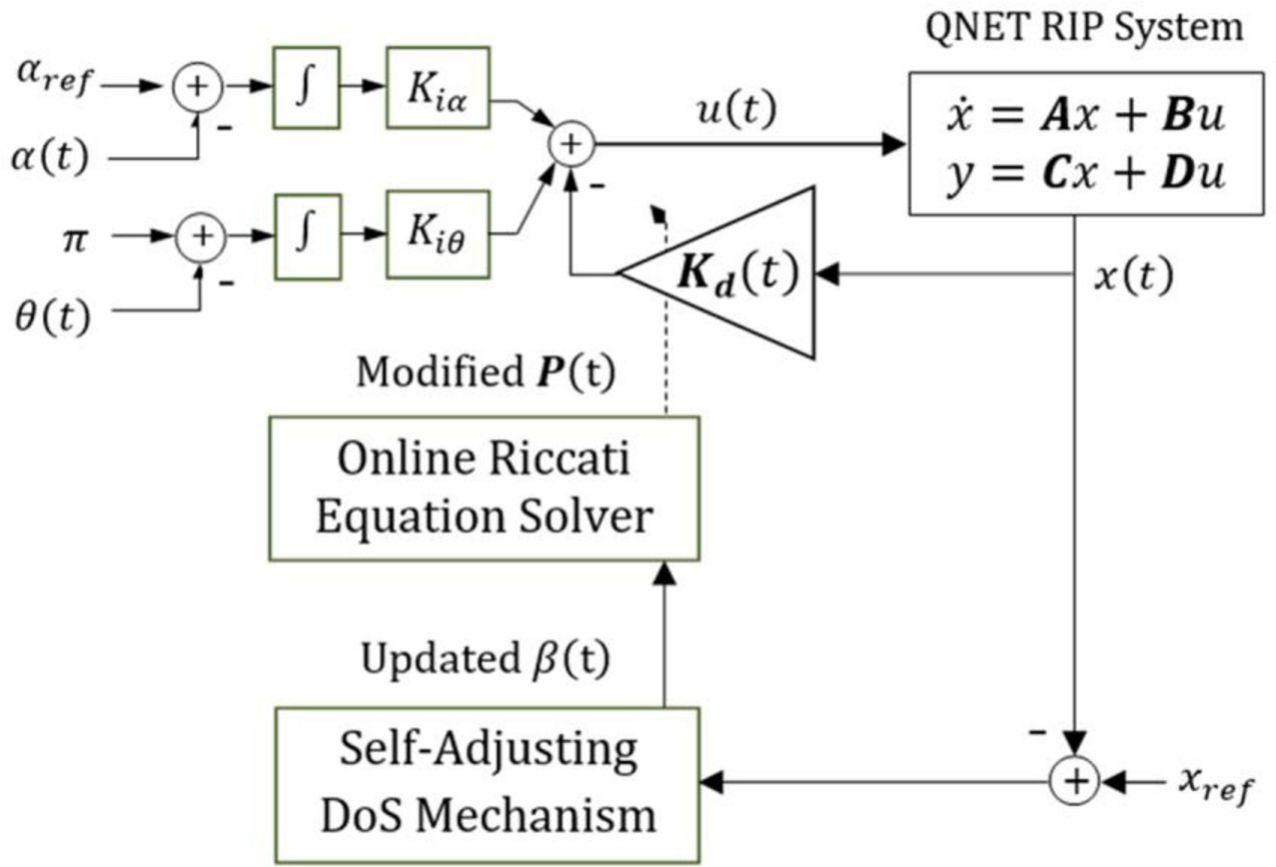
The block diagram of the ADoS-STR.

In order to constitute the adaptive control law, the value of *β* is dynamically adjusted via an online adaptation law. The proposed adaptation mechanism is formulated by using continuous nonlinear scaling functions that dynamically reconfigures the value of *β* online based on the real-time variations in the system’s cumulative position-regulation error. The cumulative position-regulation error and projected error, contributed by the pendulum’s arm and the rod, is evaluated by taking the linear combination of the individual state-error variables. The modified Riccati equation (expressed in Eq 24) uses the updated values of *β* to re-compute its solution after every sampling interval, and thus, yield a time-varying state-feedback gain vector. The structure of the STR employing the aforementioned adjustable-DoS (ADoS-STR) mechanism is illustrated in Fig 2 [64].

The online adaptation law for *β* is formulated by using a pre-calibrated continuous Hyperbolic-Secant-Function (HSF) that depends on the weighted sum of state-error variables [64]. The HSF is chosen because its waveform is continuous, bounded, and even-symmetric. The shape of HSF’s waveform is calibrated according to the following rationale [64].

The magnitude of *β* is enlarged when the state-error magnitudes increase in order to place the eigenvalues farther from the imaginary-axis. This arrangement yields stronger damping against overshoots and quickly reverses the direction of response.

1. The magnitude of *β* is reduced when the state-error magnitudes decrease in order to place the eigenvalues closer to the imaginary-axis. This allows the response to settle naturally (and smoothly).

These characteristics yield rapid convergence with strong damping against oscillations without contributing large actuating torques under the influence of bounded exogenous disturbances. The proposed HSF is formulated as follows.

$$\beta (t)=\beta_{max}-[(\beta_{max}-\beta_{min})\times \mathrm{sech}\left(z(t)\right)]$$ (27)

**Fig 3:**
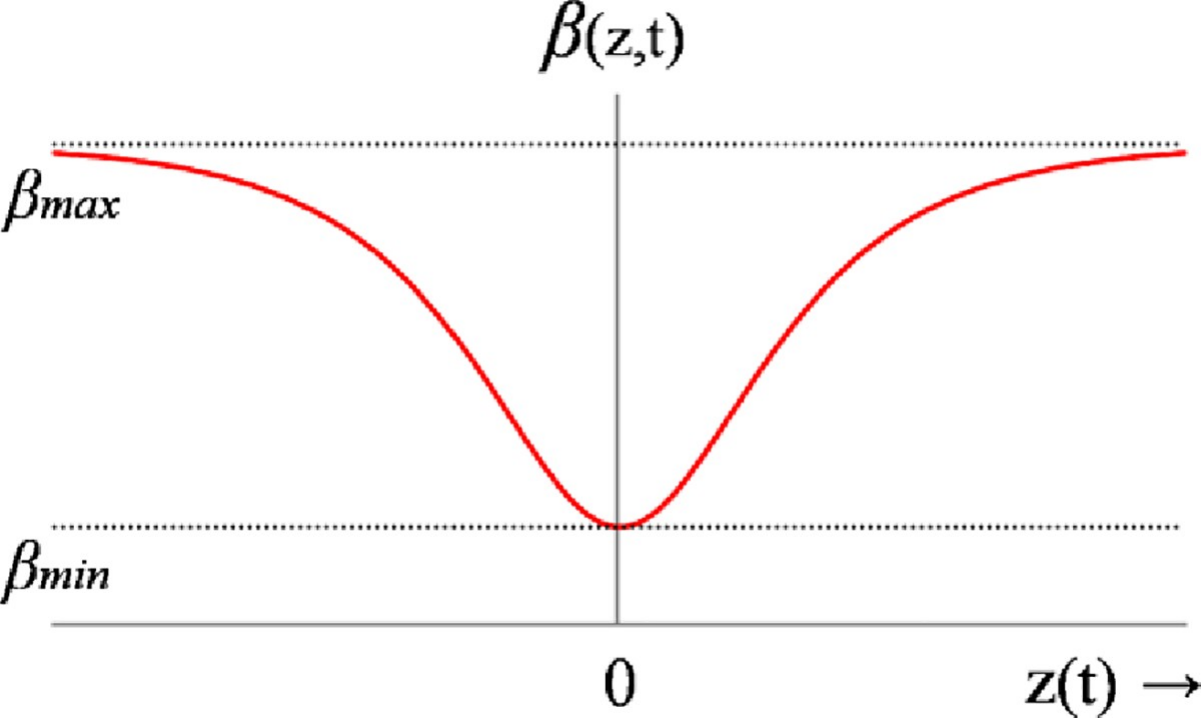
The waveform of hyperbolic-secant-function.

$such\;that,\;z(t)=\sigma_{1}e_{\alpha }(t)+\sigma_{2}e_{\theta }(t)+\sigma_{3}{\dot{e}}_{\alpha }(t)+\sigma_{4}{\dot{e}}_{\theta }(t)$ where, sech(.) represents the HSF, *β*_*min*_ and *β*_*max*_ represent the minimum and maximum limits of the HSF, *z*(*t*) is the weighted sum of all state-error variables in real-time, and the parameters *σ*_1_, *σ*_2_, *σ*_3_, and *σ*_4_ are the preset weights linked with each state-error variable in *z*(*t*). The waveform of the weight-adjusting function is shown in Fig 3.

The inclusion of the four state-error variables in the computation of *z*(*t*) informs the adaptation law regarding the effect of the disturbance on the system’s behavior. This self-reasoning capability improves the controller’s adaptability. To acquire the proposed self-reasoning capability, “positive” weights are selected for each state-error variable in *z*(*t*). Hence, when the state-responses diverge from the reference, the positive weights promote an increment in the magnitude of *z*(*t*) owing to the same polarities of the classical error variables and their derivatives in this phase. Conversely, when responses revert and approach the reference, the positive weights allow a decrement in the magnitude of *z*(*t*) owing to the opposite polarities of the classical error variables and their derivatives in this phase. This arrangement enhances the controller’s flexibility and ensures a stiff damping control effort under large error conditions to quickly attenuate the oscillations, and a softer control effort under small error conditions. The parameters are selected by minimizing *J*_*e*_ to improve the reference-tracking and disturbance-rejection behavior. The tuned parameters are recorded in Table 2.

**Table 2:** Parameter selection of the HSF for the ADoS-STR mechanism.

Parameter symbol	Parameter Range	Tuned value
*β*_*min*_	[0, 10]	0.311
*β*_*max*_	[0, 10]	0.716
*σ*_1_	[0, 100]	7.82
*σ*_2_	[0, 100]	23.37
*σ*_3_	[0, 100]	2.19
*σ*_4_	[0, 100]	6.73

### 4.2. Adjustable control-weighting-factor

In the LQR problem, the control-weighting-factor (*ρ*) steers the control input trajectory. The selection of *ρ* makes a compromise between the system’s position-regulation behavior and control energy expenditure [22]. A small value of *ρ* increases the controller’s robustness against disturbances but also induces highly discontinuous control activity. On the contrary, a large value of *ρ* limits the system’s control activity under disturbance conditions which inevitably degrades the position-regulation and transient-recovery behavior [65]. Hence, the fixed value of *ρ* renders the overall control mechanism uneconomical under rapidly changing operating conditions [66, 67]. On one hand, it applies superfluous control force under small error conditions. On the other hand, it contributes to inadequate control resources under transient disturbances. A viable solution is to adaptively modulate the *ρ* in LQR’s QPI, while keeping the coefficients of ***Q*** matrix fixed at their prescribed values, as shown below.


Q=diag(32.852.26.12.5),R(t)=ρ(t) (28)

The idea is to smoothly slide the factor *ρ* across a continuous surface so that the control profile can be flexibly manipulated to minimize the reference-tracking error and to maintain a reasonable control-input economy throughout the operating regime. This arrangement automatically relocates the eigenvalues of the closed-loop system to effectively compensate for the disturbances. With the modification, ***R***(*t*) = *ρ*(*t*), incorporated in the nominal LQR procedure, the QPI is revised as follows.


$$J_{lq}^{*}=\frac{1}{2}\int_{0}^{\infty }{x(t)}^{T}Qx(t)+{u(t)}^{T}R(t)u(t)dt$$ (29)

The modified Riccati Equation is expressed in Eq 30.


$$A^{T}P(t)+P(t)A-P(t)B{R(t)}^{-1}B^{T}P(t)+Q=0$$ (30)

The gain vector is re-computed online as follows.


$$K_{c}(t)={R(t)}^{-1}B^{T}P(t)$$ (31)

The time-varying gain vector, ***K***_***c***_(*t*), delivers the following STR control law.


$$u(t)=-K_{c}(t)x(t)+K_{i}\varepsilon (t)$$ (32)

**Fig 4:**
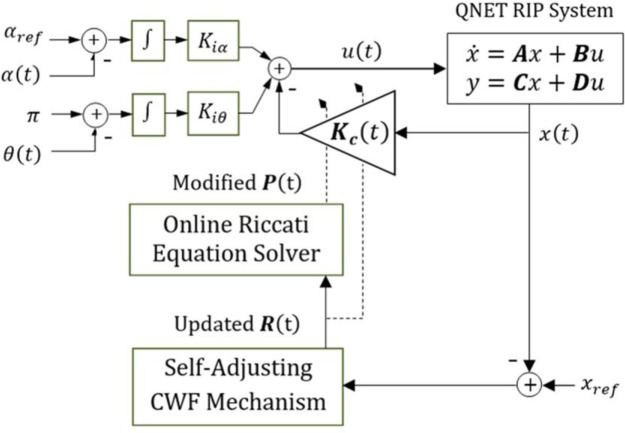
The block diagram of the ACWF-STR.

The STR equipped with the adjustable-control-weighting-factor (or ACWF) is denoted as ACWF-STR in this research [68]. Its block diagram is shown in Fig 4. The ACWF-STR yields an asymptotically-stable control behavior, as long as *ρ*(*t*)>0.

The proposed STR is implemented by augmenting the baseline LQR with a reconfiguration module that self-adjusts the value of *ρ* as a pre-calibrated nonlinear scaling function of state-error variables. The following meta-rules are used to formulate the proposed reconfiguration module [68].

1. Under small error conditions (or equilibrium state), the value of *ρ* is enlarged to allow for position-regulation with minimal control input expenditure.
2. Under large error conditions (or disturbance state), the value of *ρ* is proportionally reduced to deliver a tighter control effort to efficiently reject the disturbances.
3. If the control-input inflates drastically under the influence of bounded disturbances, the variation-rate of *ρ* is reduced to economize the control effort and limit the peak servo requirements.

With these qualities, the module dynamically restructures the control procedure to enhance the system’s response speed, strengthen its damping against oscillations, and ensure optimum allocation of control resources under exogenous disturbances. The HSF is used to ensure smooth transitions in the value of *ρ* as the operating conditions change [64]. The linear combination of the real-time state-error variables is used as the input to the HSF which aids in diagnosing the occurrence (and impact) of the exogenous disturbances. The feature dictated by the third meta-rule prevents the RIP’s DC motor from getting saturated while maintaining a reasonable response-speed and damping against oscillations [68]. This feature is incorporated in the HSF-based adaptation law by means of an auxiliary control-input-dependent function. The proposed ACWF adaptation law is formulated as follows.


$$\rho (t)=\rho_{min}+[(\rho_{max}-\rho_{min})\times \mathrm{sech}\left(\gamma (u,t)\times z(t)\right)]$$ (33)

**Fig 5:**
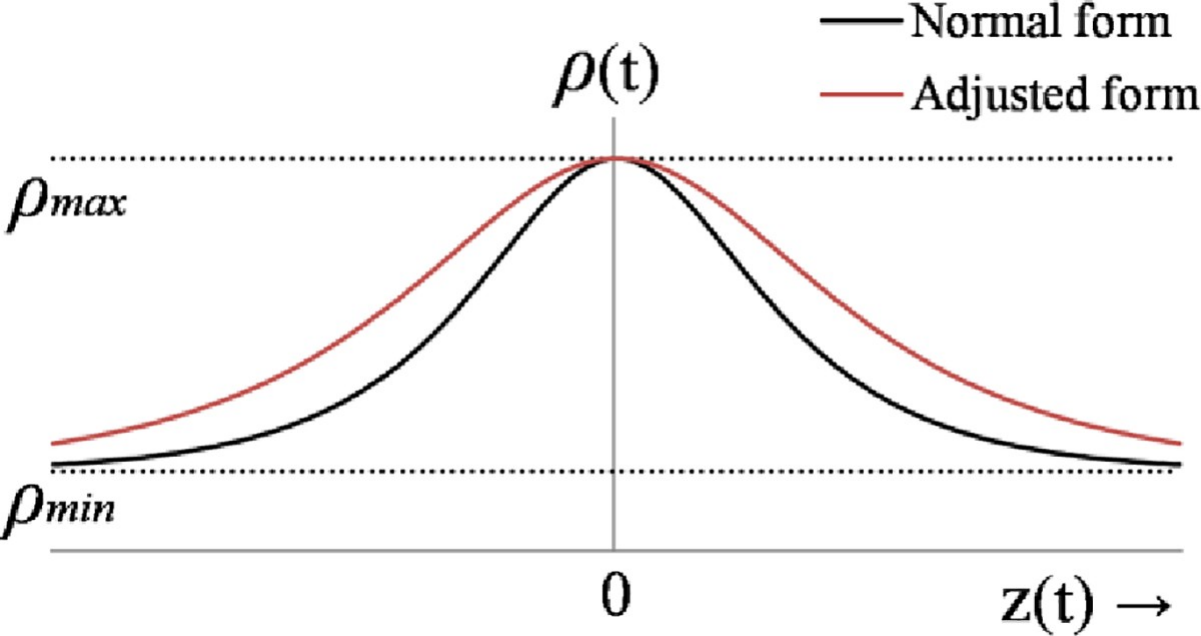
Automatic adjustment in the HSF waveform for the ACWF mechanism.

$such\;that,\;\gamma (u,t)=\gamma_{o}\times \left(\omega +\frac{1-\omega }{1+{\left|\eta u(t)\right|}^{\psi }}\right)$ where, *ρ*_*max*_ and *ρ*_*min*_ represent the upper and lower bounds of the HSF, *μ*_*c*_ is the preset variation-rate of the HSF, *z*(*t*) is the same state-error-driven variable as shown in Eq 27 [64], and *γ*(*u*, *t*) is the control-input-dependent self-adjusting variance of the HSF. The function *γ*(*u*, *t*) is specifically designed and implanted in the adaptation-law to realize the third meta-rule. The augmentation of *γ*(*u*, *t*) dynamically adjusts the variance of the adaptation law to maintain the controller’s robustness without contributing highly discontinuous control activity. The shape of the HSF waveform is adjusted, under large servo requirements, as shown in Fig 5.

The parameter *γ*_*o*_ is the basic variance of the function, *ω* is the positive constant between [0, 1] that presets the lower bound of the variance, *η* is the positive weight of *u*(*t*), and *ψ* is the positive fractional exponent of the scaled *u*(*t*) that prevents the self-adjustment at smaller control signals. The aforementioned parameters are tuned offline by iteratively minimizing *J*_*e*_. The selected values of these parameters are recorded in Table 3 [68].

**Table 3:** Parameter selection of the HSF for the ACWF-STR mechanism.

Parameter symbol	Parameter Range	Tuned value
*ρ*_*min*_	[0, 10]	1.04
*ρ*_*max*_	[0, 10]	0.21
*γ*_*o*_	[0, 10]	9.18
*ψ*	[0, 10]	1.52
*η*	[0, 1]	0.08
*ω*	[0, 1]	0.25

### 4.3. Adjustable swfs using error-phase observers

This section presents another practical adaptive control scheme that self-tunes the LQR gains by adaptively modulating all the state weighting-factors associated with the QPI [57].

For the under-actuated systems, the degrees-of-freedom to be stabilized are greater than the rank of ***R*** which makes it quite hard to establish a correlation between *ρ* and the state-variables [58]. However, the coefficients of the state-weighting-matrix ***Q*** (denoted as *q*_*x*_) hold a one-to-one correspondence with the respective state-variables. This arrangement provides a pragmatic approach to dynamically adjust the values of *q*_*x*_ online. Apart from obviating the necessity to tune and preset the state-weighting-factors based on a specific performance criterion, this approach increases the degree-of-freedom of the controller design [58]. Each weighting-factor is dynamically adjusted by using pre-calibrated nonlinear functions that are driven by the corresponding state-error variables of the system, as shown in Eq 34.


$$Q(t)=diag(q_{\alpha }(t)\;q_{\theta }(t)\;q_{\dot{\alpha }}(t)\;q_{\dot{\theta }}(t)),R=1$$ (34)

The control weighting-factor is preset to unity to maintain an economical control activity. The time-varying state weighting-matrix, ***Q***(*t*), is used to modify the solution of the Matrix-Riccati-Equation, after every sampling interval, as shown below [17].


$$A^{T}P(t)+P(t)A-P(t)BR^{-1}B^{T}P(t)+Q(t)=0$$ (35)

The updated ***P***(*t*) re-computes the state-feedback gains online by using the following update law.


$$K_{s}(t)=R^{-1}B^{T}P(t)$$ (36)

The STR equipped with adjustable State-Weighting-Factors (SWF) is shown below.


$$u(t)=-K_{s}(t)x(t)+K_{i}\varepsilon (t)$$ (37)

**Fig 6:**
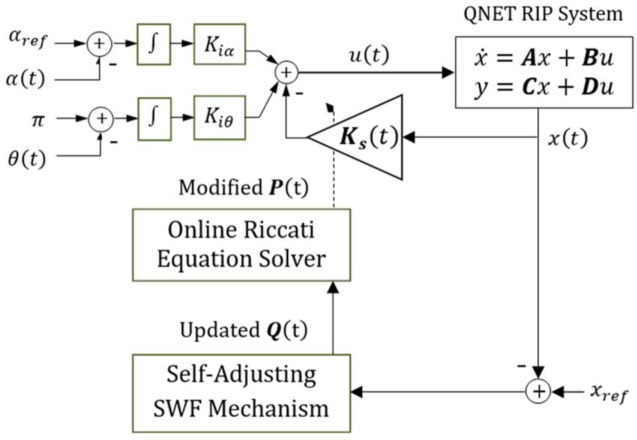
The block diagram of the ASWF-based STR scheme.

The block diagram of SWF-STR is shown in Fig 6. The following Lyapunov function is used to verify the asymptotic stability of the SWF-STR architecture [17].


$$V(t)={x(t)}^{T}P(t)x(t)>0,\;for\;x(t)\neq 0$$ (38)

The first-derivative of *V*(*t*) is expressed as follows.


$$\dot{V}(t)={x(t)}^{T}(\dot{P}(t)+A^{T}P(t)+P(t)A-P(t)BR^{-1}B^{T}P(t))x(t)$$ (39)

The term $\dot{P}(t)$ approaches to zero in an infinite horizon control problem [35]. Thus, the simplified expression of $\dot{V}(t)$ reduces to Eq 40.


$$\dot{V}(t)=-{x(t)}^{T}Q(t)x(t)<0$$ (40)

The expression of first-derivative is negative-definite as long as ***Q***(*t*)>0, which justifies the stability of the proposed STR.

This adaptation law relies upon the “phase” of the system’s state-response(s) to adaptively tune the state-weighting-factors [53]. The baseline weight-adjusting functions are implemented via pre-calibrated HSFs that depend on the variations in the magnitude of the classical state-error and phase of the state-response. These HSFs are retrofitted with an auxiliary phase-observer that accurately “deduces” and informs the adaptation mechanism regarding the movement of the state-response (away or towards the reference) based only on the instantaneous polarities of the classical state-error and the state-error-derivative variables [54]. The “phase” information is also used to automatically “mutate” the shape of each HSF waveform. This synthetic self-deduction and self-mutation capability significantly enhances the robustness of the adaptive control procedure against exogenous disturbances; thus, making it highly suitable for damping control applications. The following qualitative rules are used to constitute the online adaptation mechanism [53].

1. When the response is diverging from the reference, the values of *q*_*x*_ are inflated to apply a stiff control effort which damps the overshoots and reverses the direction of response.
2. When the response is converging to the reference, the values of *q*_*x*_ are reduced to apply a soft control effort which allows the response to settle (naturally) with minimum fluctuations.

**Fig 7:**
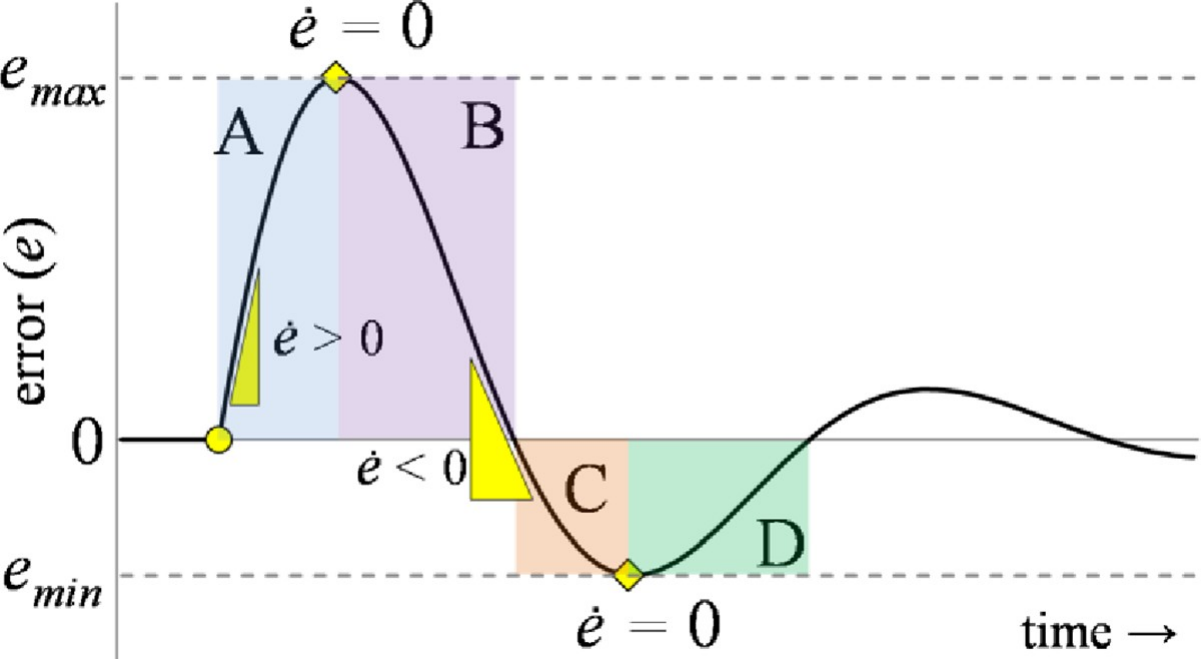
Error profile of an arbitrary under-damped system.

These characteristics induce rapid transits in the response with strong damping against oscillations while suppressing the peak servo requirements. However, this rationale requires precise information regarding the phase (direction of motion) of the response to restructure the control procedure. Consider the time-domain error profile of an arbitrary under-damped system, shown in Fig 7, under the influence of a bounded disturbance.

The error profile is divided into four phases; A, B, C, and D. Each phase represents a distinct operating condition that is addressed individually to attain the best control effort. The polarities of error and error-derivative are the same when the response is deviating from the reference (phases A and C). The polarities of error and error-derivative are opposite when the response is converging to reference (phases B and D) [53, 54]. In lieu of this state-error behavior, the phase is observed as follows [69].

$$m_{x}=step(e_{x}(t)\times {\dot{e}}_{x}(t))$$ (41)

where, and *m*_*x*_ is a step(.) function that yields a “zero” if its internal product yields a negative value and a “one” if the internal product yields a positive value, and ‘*x*’ denotes the state-variable being considered. This phase-observer is embedded within the structure of a state-error-dependent HSF to alter the waveform’s shape as the state-error changes [69]. The proposed self-mutating HSF is given in Eq 42 [59].

$$q_{x}(t)={m_{x}a}_{x}-(b_{x}-(1-m_{x})a_{x})\times \mathrm{sech}\left(\gamma_{x}e_{x}(t)\right)$$ (42)

where, *a*_*x*_ and *b*_*x*_ are the positive upper and lower bounds of each function such that *a*_*x*_≥*b*_*x*_ to ensure *q*_*x*_(*t*)≥0, and *γ*_*x*_ represents the variance of each function. The proposed HSF complies with the aforementioned meta-rules. Each weight-adjusting function is augmented with its corresponding Boolean operator, *m*_*α*_ or *m*_*θ*_.

**Fig 8:**
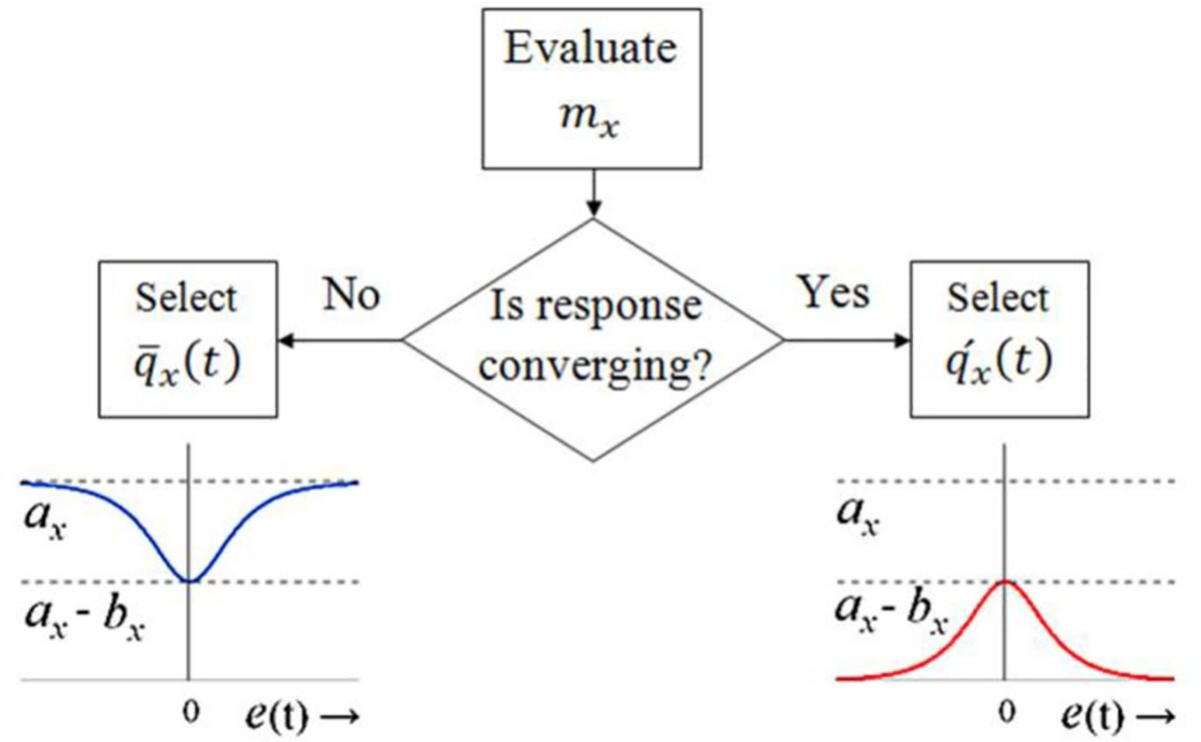
Self-mutation scheme for weight-adjusting functions.

The logical rules governing the self-mutation of *q*_*x*_(*t*) are defined in Table 4. The mutation scheme is illustrated in Fig 8 [59]. In phases A and C, the response deviates from the reference. Since the error and error-derivative variables have the same polarities, the Boolean-setting of *m*_*x*_ = 1 selects the growing function of the form ${\bar{q}}_{x}(t)$. This setting delivers a tight control effort to damp the overshoot (or undershoot). In phases B and D, the response converges to reference. The error and error-derivative variables have opposite polarities which lead to the Boolean-setting of *m*_*x*_ = 0. This setting contributes a relatively gentle control effort to allow for a quick yet smooth settlement of the response.

**Table 4:** Mutation of weight-adjusting functions.

Phase	*e*_*x*_	${\dot{e}}_{x}$	Response	*m*_*x*_	Mutation of *q*_*x*_(*t*) waveform
A	> 0	> 0	Diverging	+1	${\bar{q}}_{x}(t)=a_{x}-(b_{x}\times \mathrm{sech}\left(\gamma_{x}e_{x}(t)\right))$
B	> 0	< 0	Converging	0	$\overset{´}{q_{x}}(t)=(a_{x}-b_{x})\times \mathrm{sech}\left(\gamma_{x}e_{x}(t)\right)$
C	< 0	< 0	Diverging	+1	${\bar{q}}_{x}(t)=a_{x}-(b_{x}\times \mathrm{sech}\left(\gamma_{x}e_{x}(t)\right))$
D	< 0	> 0	Converging	0	$\overset{´}{q_{x}}(t)=(a_{x}-b_{x})\times \mathrm{sech}\left(\gamma_{x}e_{x}(t)\right)$

**Fig 9:**
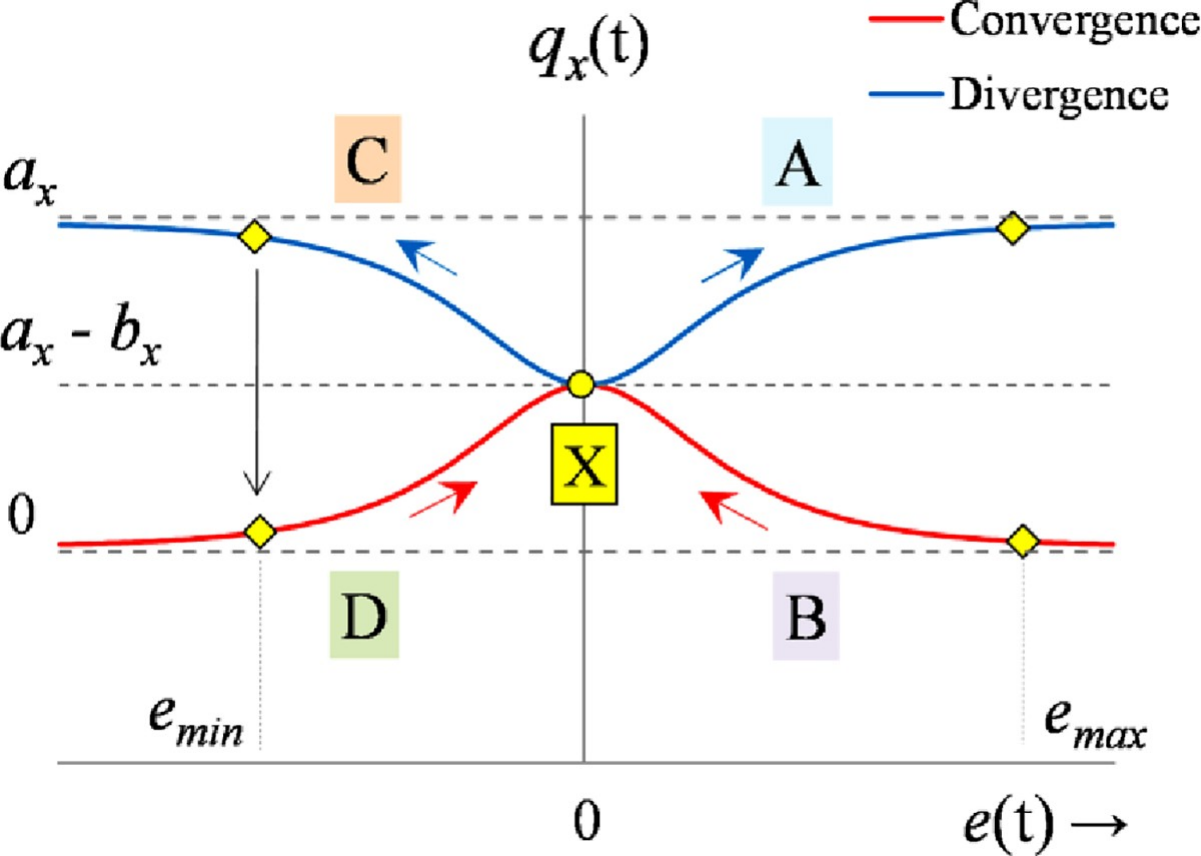
Variation rules for weighting coefficients in every phase.

With the commissioning of the phase-observer, the weight-adjusting function(s) autonomously reconfigure their waveforms as illustrated in Fig 9 [53]. The proposed augmentation strengthens the controller’s disturbance-rejection capability by autonomously transforming the growing behaviour of the waveform into a decaying behaviour as the state-response transits from divergence phase to convergence phase, and vice-versa. The self-mutating error-phase-based HSFs are formulated as follows [59].


$$q_{\alpha }(t)={m_{\alpha }a}_{\alpha }-\left((b_{\alpha }-(1-m_{\alpha })a_{\alpha })\times \mathrm{sech}\left(\gamma_{\alpha }e_{\alpha }(t)\right)\right)$$ (43)

$$q_{\theta }(t)={m_{\theta }a}_{\theta }-\left((b_{\theta }-(1-m_{\theta })a_{\theta })\times \mathrm{sech}\left(\gamma_{\theta }e_{\theta }(t)\right)\right)$$ (44)

$$q_{\dot{\alpha }}(t)={m_{\alpha }a}_{\dot{\alpha }}-\left((b_{\dot{\alpha }}-(1-m_{\alpha })a_{\dot{\alpha }})\times \mathrm{sech}\left(\gamma_{\dot{\alpha }}e_{\alpha }(t)\right)\right)$$ (45)

$$q_{\dot{\theta }}(t)={m_{\theta }a}_{\dot{\theta }}-\left((b_{\dot{\theta }}-(1-m_{\theta })a_{\dot{\theta }})\times \mathrm{sech}\left(\gamma_{\dot{\theta }}e_{\theta }(t)\right)\right)$$ (46)

$such\;that,\;m_{\alpha }=step(e_{\alpha }(t)\times {\dot{e}}_{\alpha }(t)),\;m_{\theta }=step(e_{\theta }(t)\times {\dot{e}}_{\theta }(t))$

The hyper-parameters associated with each weight-adjusting function are tuned by iteratively minimizing *J*_*e*_ to yield strong damping control. The tuned parameters are shown in Table 5 [59].

**Table 5:** Parameter selection of the error-phase-dependent HSFs.

Parameter symbol	Parameter range	Identified value
*a*_*α*_	[0, 500]	351.60
*b*_*α*_	[0, 500]	168.45
*a*_*θ*_	[0, 500]	200.00
*b*_*θ*_	[0, 500]	95.55
$a_{\dot{\alpha }}$	[0, 50]	10.15
$b_{\dot{\alpha }}$	[0, 50]	5.42
$a_{\dot{\theta }}$	[0, 50]	8.22
$b_{\dot{\theta }}$	[0, 50]	4.36
*γ*_*α*_	[0, 50]	6.10
*γ*_*θ*_	[0, 50]	19.88
$\gamma_{\dot{\alpha }}$	[0, 50]	2.11
$\gamma_{\dot{\theta }}$	[0, 50]	5.95

The adapted values of *q*_*x*_(*t*) remain positive throughout the operating regime, which ensures the system’s stability. The STR equipped with the self-mutating error-phase-dependent HSFs is denoted as the “EP-STR” [59].

### 4.4. Adjustable SWFs using error-magnitude observers

The proposed scheme dynamically updates the state-feedback gains, after every sampling interval, by adaptively modulating the state-weighting-factors as well as the control-input weighting factor associated with the QPI, concurrently, by using online state-error dependent expert self-tuning mechanism(s) [57]. This arrangement is beneficial because it indirectly alters the state-feedback gains by harnessing the full potential of the proposed hierarchical adaptive LQR scheme by dynamically adjusting all the user-specified constituent weighting-factors of the Riccati equation.

It enhances the adaptability of control procedure to realize the environmental indeterminacies and flexibly steer the control profile to compensate for the consequent parametric variations. The weighting-matrices containing the self-adjusting coefficients are represented as follows.


$$Q(t)=diag(q_{\alpha }(e_{\alpha },t)\;q_{\theta }(e_{\theta },t)\;q_{\dot{\alpha }}({\dot{e}}_{\alpha },t)\;q_{\dot{\theta }}({\dot{e}}_{\theta },t)),R(t)=\rho (e_{\alpha },e_{\theta },t)$$ (47)

The value of ***R*** is maintained at unity to economize the control energy expenditure. The rationale and the methodology used to formulate the state-error dependent online self-tuning mechanism(s) for the state-weighting-factors is discussed in the following discussions. The restructured Riccati Equation is expressed in Eq 48.


$$A^{T}P(t)+P(t)A-P(t)B{R(t)}^{-1}B^{T}P(t)+Q(t)=0$$ (48)

The Riccati Equation yields a time-varying solution, ***P***(*t*), after every sampling instant. The self-adjusting state-feedback gain vector is calculated by using Eq 49.


$$K(t)={R(t)}^{-1}B^{T}P(t)$$ (49)

The proposed STR law is defined as follows.


$$\hat{u}(t)=-K(t)x(t)+K_{I}\varepsilon (t)$$ (50)

This self-tuning strategy observes the real-time variations in the state-error magnitudes to dynamically adjust the weighting-factors while preserving the system’s stability throughout the operating regime. The rationale used to develop the error-magnitude observer for self-tuning control of robotic systems has been experimentally verified in the available literature [11]. It relies upon the following two meta-rules to modify the critical controller parameters [52].

1. The proportional state-weighting-factors (*q*_*α*_ and *q*_*θ*_) are inflated as the magnitude of corresponding classical state-errors tend to reduce, and vice-versa.
2. The differential state-weighting-factors ($q_{\dot{\alpha }}$ and $q_{\dot{\theta }}$) are inflated as the magnitude of corresponding state-error-derivatives tend to increase, and vice-versa.

**Fig 10:**
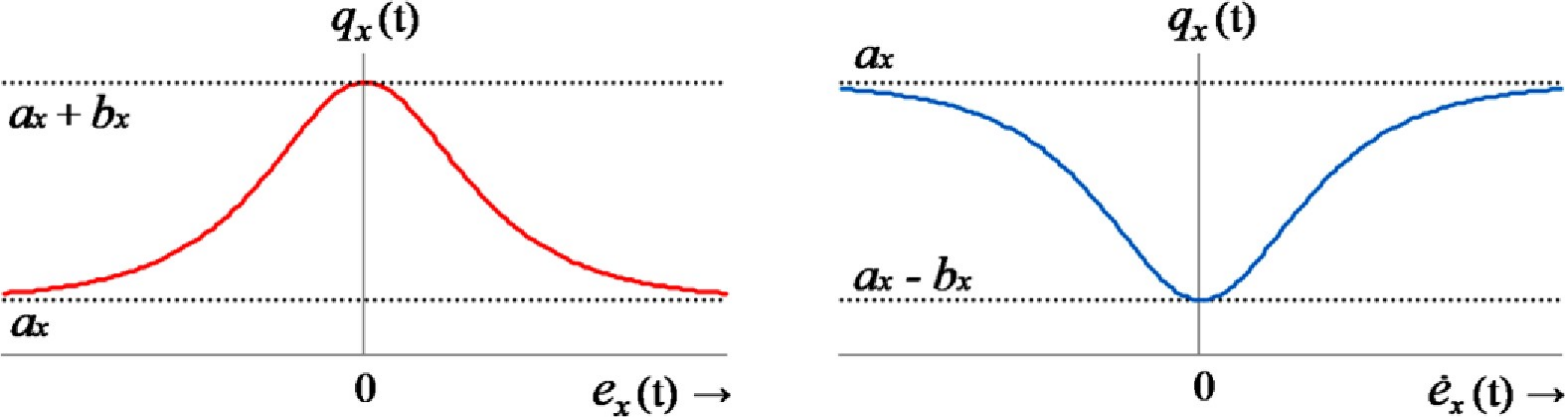
The waveforms of the proportional (left) and the differential (right) weight-adjusting functions.

Together, these characteristics dynamically reconfigures the control procedure to strengthen the system’s disturbance-compensation capability [11, 52]. To ensure a smooth transition of the weighting-factors, nonlinear scaling functions are required to be continuous, bounded, and even-symmetric. Hence, the weight-adaptation functions are implemented via partial-hyperbolic-functions (PHFs), whose shapes and forms are configured offline according to the above-mentioned qualitative rules [70]. It is to be noted that the hyperbolic secant functions and zero-mean Gaussian functions can also be used instead of the PHF to mathematically program the said adaptation law [64, 68]. The error-magnitude driven PHFs used to scale each state and control weighting-factor are formulated below [71].

$$q_{\alpha }(e_{\alpha },t)=a_{\alpha }+\frac{b_{\alpha }}{1+{\left|\gamma_{\alpha }e_{\alpha }(t)\right|}^{2}}$$ (51)

$$q_{\theta }(e_{\theta },t)=a_{\theta }+\frac{b_{\theta }}{1+{\left|\gamma_{\theta }e_{\theta }(t)\right|}^{2}}$$ (52)

$$q_{\dot{\alpha }}({\dot{e}}_{\alpha },t)=a_{\dot{\alpha }}-\frac{b_{\dot{\alpha }}}{1+{\left|\gamma_{\dot{\alpha }}{\dot{e}}_{\alpha }(t)\right|}^{2}}$$ (53)

$$q_{\dot{\theta }}({\dot{e}}_{\theta },t)=a_{\dot{\theta }}-\frac{b_{\dot{\theta }}}{1+{\left|\gamma_{\dot{\theta }}{\dot{e}}_{\theta }(t)\right|}^{2}}$$ (54)

$$\rho (e_{\alpha },e_{\theta },t)=a_{u}-\frac{b_{u}}{1+{\left|\gamma_{u}(\gamma_{\alpha }e_{\alpha }(t)+\gamma_{\theta }e_{\theta }(t))\right|}^{3}}$$ (55)

where, *a*_*x*_ and *b*_*x*_ represent the prescribed upper and lower bounds of the state-weighting functions, and *γ*_*x*_ represents the variance of the state-weighting functions. The waveforms of the weighting-adjusting functions are shown in Fig 10.

A proper selection of the *γ*_*x*_ enables the controller to apply a stiffer control effort under disturbed state and a softer control effort under equilibrium state of the system. This arrangement strengthens the system’s damping against fluctuations, yields minimum-time transient recovery and renders a smoother control activity [71]. It also averts the limit-cycles contributed by static-friction during dead-zones. In this mechanism, the value of *ρ* is also adaptively modulated as a nonlinear function of the classical state-error variables. This arrangement prevents the actuator from getting saturated due to the rapid fluctuations and large overshoots in the control-input profile, without trading-off the system’s robustness, under exogenous disturbances [52]. It contributes rapid transits with strong damping against disturbances while economizing the control-energy expenditure [71].

The prescribed bounds of each hyperbolic function are carefully selected so that *q*_*x*_(.)>0 and *ρ*(.)>0, under every operating condition, to ensure an asymptotically stable control behavior. The hyper-parameters associated with each weight-adjusting function are tuned by iteratively minimizing *J*_*c*_ to attain the best position-regulation accuracy. The tuned parameters are presented in Table 6 [71]. The STR constructed via the error-magnitude driven PHFs is denoted as “EM-STR”.

**Table 6:** Parameter selection of the error-magnitude-dependent PHFs.

Parameter symbol	Parameter range	Identified value
*a*_*α*_	[0, 500]	1.85
*a*_*θ*_	[0, 500]	2.41
$a_{\dot{\alpha }}$	[0, 50]	11.05
$a_{\dot{\theta }}$	[0, 50]	10.05
*a*_*u*_	[0, 50]	2.11
*b*_*α*_	[0, 500]	408.15
*b*_*θ*_	[0, 500]	291.62
$b_{\dot{\alpha }}$	[0, 50]	10.88
$b_{\dot{\theta }}$	[0, 50]	9.97
*b*_*u*_	[0, 50]	1.11
*γ*_*α*_	[0, 50]	2.12
*γ*_*θ*_	[0, 10]	5.96
$\gamma_{\dot{\alpha }}$	[0, 10]	1.10
$\gamma_{\dot{\theta }}$	[0, 10]	3.05
*γ*_*u*_	[0, 10]	0.52

## 5. Comparative performance assessment

This section presents a detailed overview of the hardware setup, testing procedure, and comparative experimental analysis of the proposed control schemes.

### 5.1. Experimental setup

**Fig 11:**
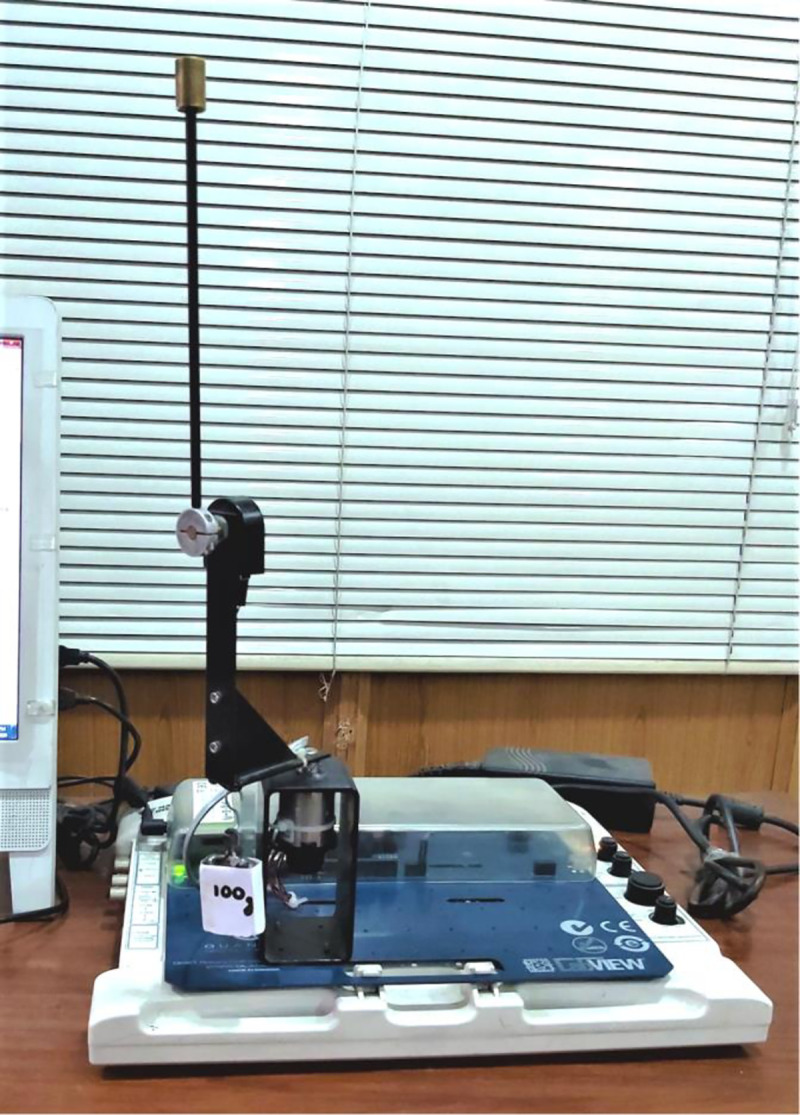
The QNET Rotary Pendulum’s hardware setup.

The proposed self-tuning control mechanisms are analyzed by conducting hardware experiments on QNET RIP hardware setup [62]. The angular displacements, *θ* and *α*, are measured in real-time by using the optical rotary encoders that are commissioned on-board the hardware setup. These encoders are installed at the pivot of the pendulum rod and with the motor’s shaft, respectively. The hardware setup uses NI-ELVIS II data-acquisition board to capture the encoder measurements and digitize them at a sampling rate of 1000 Hz [11]. The digitized measurements are then serially transmitted to the software control routine at 9600 bps. The customized control routine is digitally implemented by using the “Block Diagram” tool as well as the built-in mathematical functions available in the virtual-instrument file of the LABVIEW Software. The said software is running on a 2.0 GHz digital computer with 8.0 GB RAM. After every sampling instant, the control routine receives the updated sensor measurements, adjusts the critical controller parameters, and computes the modified control signal. The control routine uses the computer’s built-in real-time clock to plan the successive updates in weighting factors after every sampling interval. The front-end of the control software acts as a user interface that records and graphically displays the real-time variations in the state and control-input. The generated control signals are serially transmitted back to the motor driver circuit that is installed on-board the hardware setup. The driver circuit translates the incoming motor control signals into pulse-width-modulated commands that are subsequently amplified to actuate the DC motor. The DC motor and its driving circuit, commissioned on the RIP hardware setup, are durable and agile enough to handle the discontinuous control activity contributed by the proposed control schemes. The QNET Rotary Pendulum’s hardware setup is shown in Fig 11.

### 5.2. Tests and results

**Fig 12:**
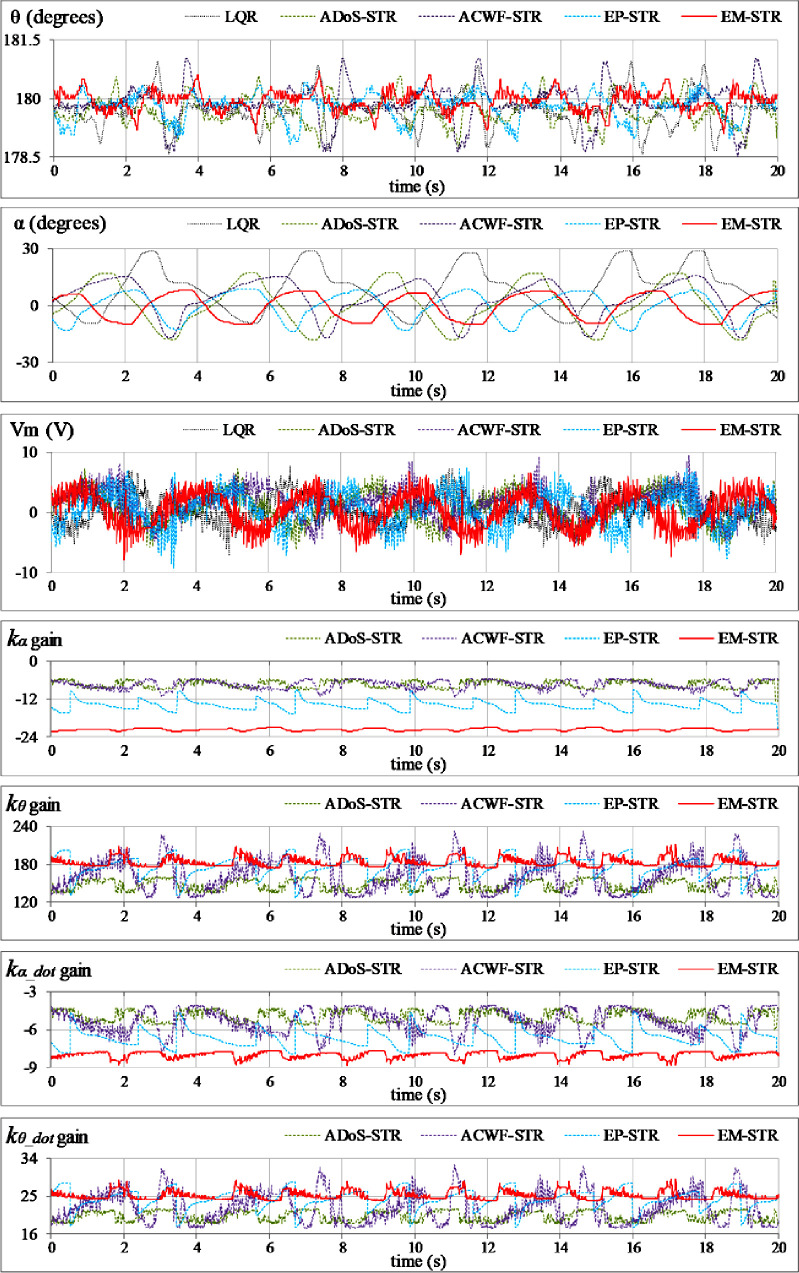
Pendulum’s response under normal conditions.

**Fig 13:**
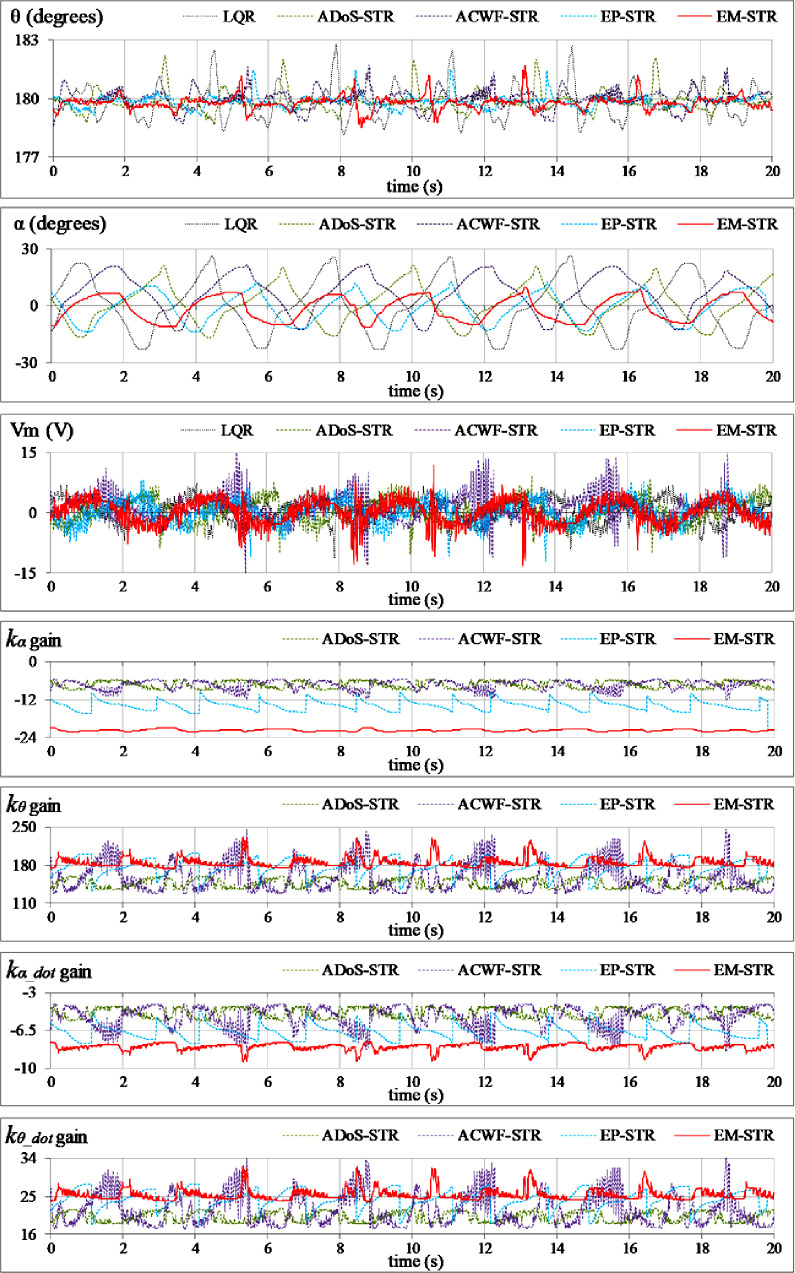
Pendulum’s response under impulsive disturbances.

**Fig 14:**
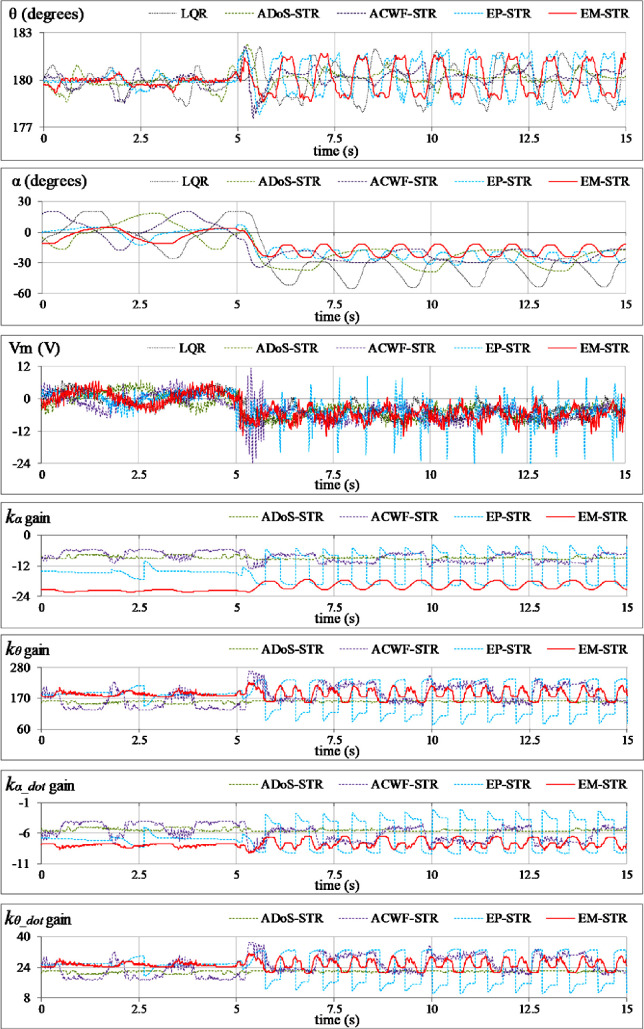
Pendulum’s response under step disturbance.

**Fig 15:**
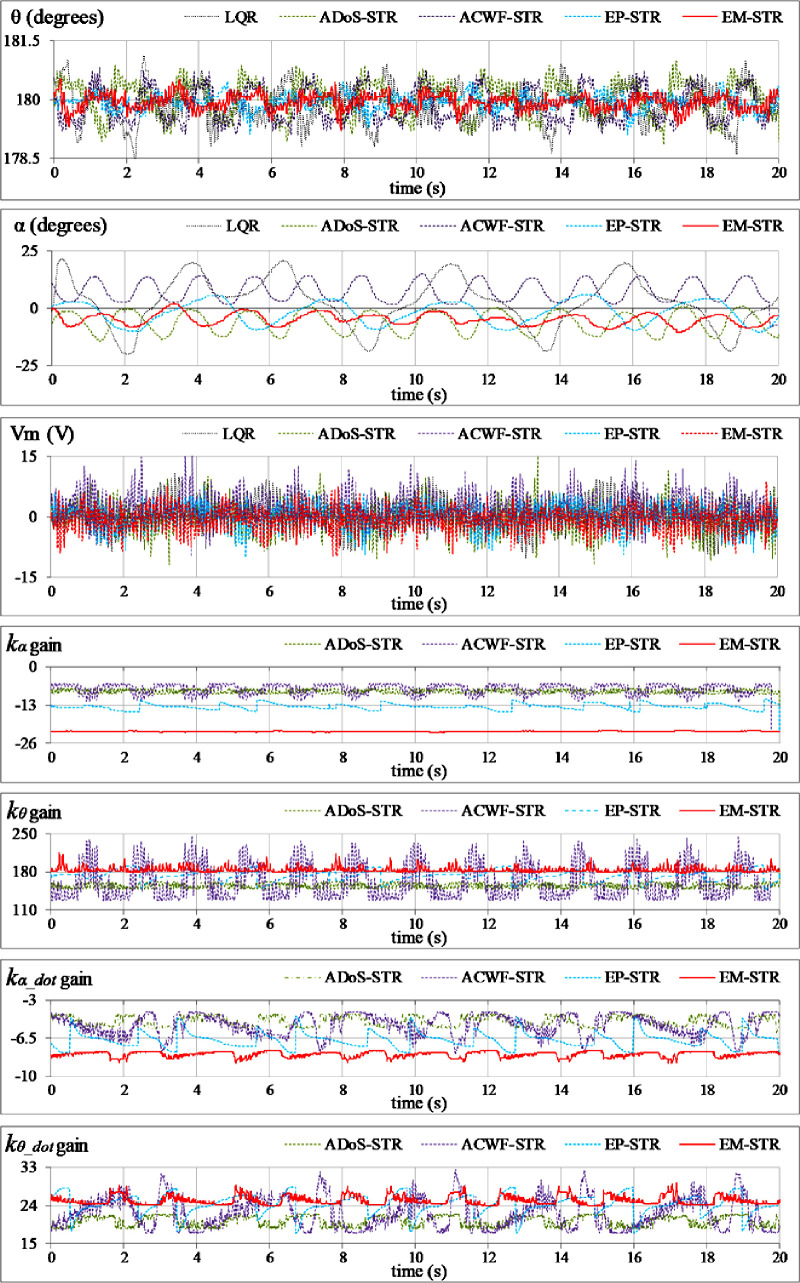
Pendulum’s response under sinusoidal disturbance.

**Fig 16:**
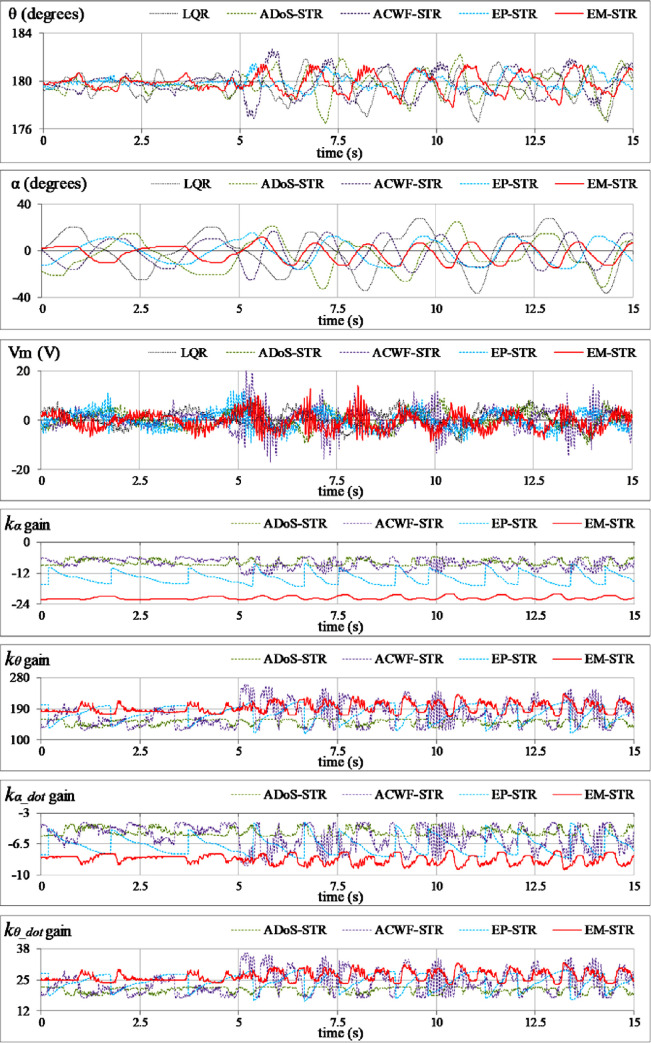
Pendulum’s response under model variation.

The position-regulation and disturbance-compensation capability of the proposed adaptive control schemes are compared by conducting five unique “hardware-in-the-loop” experiments on the QNET pendulum setup. The time-domain state and control-input variations are recorded for comparative analysis. The graphical results pertaining to *θ* and *α* are depicted in degrees (or deg.) to simplify the visual understanding. The detailed testing procedures along with the corresponding graphical results are presented as follows:

- **Reference tracking:** The position-regulation behavior of the pendulum under normal conditions is analyzed by allowing the rod and the arm to track their respective reference positions. The variations in *θ*(*t*), *α*(*t*), *V*_*m*_(*t*), and *K*(*t*) are shown in Fig 12.
- **Impulsive-disturbance compensation:** The controller’s ability to compensate the impact of bounded impulsive disturbances is examined by applying a pulse-signal in the *V*_*m*_(*t*) profile to perturb the state-response(s). The applied pulse has a time-duration and a peak-magnitude of 100.0 ms and -5.0 V, respectively. The pulse signal is injected in the control response at discrete intervals. The resulting variations in *θ*(*t*), *α*(*t*), *V*_*m*_(*t*), and and *K*(*t*) are shown in Fig 13.
- **Step-disturbance attenuation:** The controller’s ability to attenuate random exogenous torques is assessed by injecting a -5.0 V step-disturbance signal in the *V*_*m*_(*t*) profile at t ≈ 5.0 s mark. The behavior of *θ*(*t*), *α*(*t*), *V*_*m*_(*t*), and and *K*(*t*) are illustrated in Fig 14.
- **Noise suppression:** The controller’s ability to suppress the chattering and control-input ripples induced by the lumped disturbances, measurement noise, or the hysteresis contributed by the parasitic impedances in electronic components is analyzed by injecting a low-amplitude and high-frequency sinusoidal signal, *d*(*t*) = 1.5 sin(20*πt*), in the system’s control input voltage, *V*_*m*_(*t*). The time-domain profiles of *θ*(*t*), *α*(*t*), *V*_*m*_(*t*), and and *K*(*t*) are depicted in Fig 15.
- **Model-error rejection:** The controller’s ability to reject the identification errors and the real-time model variations is evaluated by changing the pendulum arm’s mass to modify the coefficients of state and input matrices of the system’s model, expressed in Section 2. This modification is realized by attaching a 0.10 kg metallic mass beneath the pendulum’s arm via a hook, as shown in Fig 11, at t ≈ 5.0 s mark. This modification abruptly changes the coefficients of the system’s model during the experiment, and thus, induces perturbations in the pendulum’s response. The behavior of *θ*(*t*), *α*(*t*), *V*_*m*_(*t*), and *K*(*t*) are illustrated in Fig 16.

### 5.3. Analysis and discussions

The quantitative analysis of the experimental results is done with the aid of the following seven Key-Performance-Indicators (KPIs):

- The root-mean-squared value of error (RMSE_x_) in the pendulum angle response(s).
- The mean-squared value of the applied DC motor voltage (MSV_m_).
- The magnitude of the peak overshoot (OS_θ_) observed in *θ*(*t*).
- The time taken by pendulum’s rod (t_set_) to settle within ±2% of the reference after a disturbance.
- The disturbance-induced angular offset in the arm’s position (α_offset_).
- The peak-to-peak amplitude of the disturbance-induced fluctuations in the arm’s position (α_pp_).
- The magnitude of peak motor voltage (V_m,p_).

The aforementioned KPIs are used as the standard performance measures in the available literature to critically analyze the position-regulation behavior, disturbance-rejection capability, and control energy requirements of the system [15, 64]. The experimental results, expressed in terms of the aforementioned KPIs, are summarized in Table 7. The proposed control schemes remain stable under every disturbance condition. The results clearly indicate that the generic LQR underperforms as compared to the adaptive controller variants in every test case. The ADoS-STR exhibits a moderately better position regulation behavior as compared to generic LQR. Its control-input economy is relatively better than ACWF-STR in every test-case. The ACWF-STR manifests significant improvement in the robustness but also renders highly discontinuous control activity which contributes to chattering in the response of *θ*(*t*).

**Table 7:** Summary of experimental results.

Test	KPI	Controller Types				
		LQR	ADoS-STR	ACWF-STR	EP-STR	EM-STR
A	RMSE_*θ*_ (deg.)	0.50	0.48	0.47	0.38	0.27
	RMSE_*α*_ (deg.)	14.64	11.08	9.97	7.23	6.79
	MSV_m_ (V^2^)	6.33	6.75	8.28	7.70	7.98
B	RMSE_*θ*_ (deg.)	0.80	0.53	0.47	0.35	0.40
	|OS_θ_| (deg.)	2.81	2.19	1.75	1.49	1.67
	t_rec_ (s)	0.77	0.67	0.62	0.53	0.59
	RMSE_*α*_ (deg.)	14.04	9.92	11.93	7.80	6.89
	MSV_m_ (V^2^)	9.14	8.21	12.79	8.04	9.94
	V_m,p_ (V)	-11.38	-10.19	-15.76	-11.97	-13.68
C	RMSE_*θ*_ (deg.)	0.97	0.53	0.52	1.05	0.84
	RMSE_*α*_ (deg.)	32.23	22.90	21.16	19.03	16.25
	*α*_offset_ (deg.)	-39.05	-27.50	-23.13	-21.25	-17.75
	*α*_pp_ (deg.)	28.50	20.52	13.25	12.89	13.24
	MSV_m_ (V^2^)	25.02	24.89	31.47	31.73	31.34
D	RMSE_*θ*_ (deg.)	0.48	0.45	0.47	0.21	0.21
	RMSE_*α*_ (deg.)	10.93	7.65	8.73	5.32	5.39
	MSV_m_ (V^2^)	13.18	13.44	17.65	8.10	8.53
E	RMSE_*θ*_ (deg.)	1.12	1.04	1.03	0.55	0.78
	RMSE_*α*_ (deg.)	16.84	14.22	11.39	9.54	7.65
	MSV_m_ (V^2^)	11.29	10.72	17.69	11.83	11.81

The EP-STR exhibits a time-optimal behavior as compared to ACWF-STR and ADoS-STR. Apart from contributing enhanced disturbance-rejection; it delivers better control-input efficiency than other STR variants while maintaining the system’s asymptotic-stability throughout the operating regime. However, its time-domain performance is inferior to that of EM-STR, especially under the testing scenarios Test A, C, and E. The EM-STR demonstrates significant enhancement in the disturbance compensation capability and position-regulation accuracy as compared to the EP-STR. However, amid transient disturbances, the EM-STR consumes relatively large control energy and exhibits large peaks in the control voltage profile. A concise qualitative analysis of the performances of the proposed STR variants is summarized as follows:

In **Test-A**, the RIP exhibits the largest deviations in the angular responses under the influence of LQR. The deviations in the responses of *θ* and *α* progressively reduce as the nominal LQR is retrofitted with enhanced adaptation mechanisms. The ASWF-adapted EM-STR shows optimum position-regulation accuracy with minimum reference-tracking error, minimum chattering, and reasonably low control-energy consumption as compared to other adaptive controller variants (except for EP-STR). The EP-STC shows the second-best position-regulation performance and the best control energy expenditure amongst the other STR variants. The pendulum response of ADoS-STR shows a fixed offset of 0.3 deg. from the vertical reference throughout the experimental trial. The ACWF-STR shows persistent chattering in *θ*(*t*).

In **Test-B**, the ILQR demonstrates the slowest transient-recovery and insufficient damping against the impulsive disturbances. It demonstrates the largest magnitude of the peak overshoot in the pendulum’s response, which is followed by persistent steady-state oscillations. The ACWF-STR continues to exhibit a highly discontinuous control activity. The EP-STR exhibits minimum transient-recovery time to effectively attenuate the oscillations and shows minimum OS_θ_ while attenuating the impulsive disturbances. The EP-STR consumes minimum average control-input energy (MSV_m_). Its peak servo requirements are also much smaller than that of EM-STR. The EM-STR shows minimum steady-state fluctuations upon convergence, owing to the augmentation of the phase-based self-learning capability of the controllers.

In **Test-C**, the step-disturbance permanently displaces the arm from its reference position. The LQR manifests the largest post-disturbance displacement in the arm’s position and large oscillations in the rod. The intermediate STR variants demonstrate moderately better transient-recovery behavior with reasonable damping against the oscillations. The EM-STR, however, effectively suppresses the influence of the applied step-disturbance by contributing minimum RMSE and offset in the nominal positions of the pendulum and the arm, respectively. It exhibits the minimum *α*_*offset*_ and the minimum peak-to-peak amplitude of the oscillations in the pendulum’s responses, *θ*(*t*). Furthermore, EM-STR contributes a slightly better control-input economy as compared to EP-STR. The ADoS-STR exhibits the most economical control-input behavior in this test-case.

In **Test D**, the EP-STR effectively attenuates the ripples in the response caused by the noise. Despite the noise, the EP-STR controlled system manages to regulate the pendulum at the desired reference position(s) with minimal RMSE and minimum control voltage requirements. The EM-STR exhibits the second-best time-domain behavior, in terms of the control-energy expenditure and position-regulation accuracy.

In **Test-E**, the EM-STR again surpasses other STR variants compared in this article. It robustly compensates the perturbations induced by the modeling-error by delivering strongly damping against the oscillations in the state-responses, and thus, minimizing the reference-tracking error as well as the control energy consumption. The EM-STR effectively attenuates the peak-to-peak amplitude of the post-disturbance oscillations in the state-responses. The control activity of the AI-STC controlled system is relatively smoother than the EP-STR variants. The EP-STR exhibits the minimum RMSE in the pendulum’s angular profile, *θ*(*t*). The ADoS-STR exhibits the most economical control-input behavior in this test-case.

From a functional point of view, the state-feedback gains associated with EM-STR respond and adapt to the real-time state-variations relatively quickly. Unlike other STR variants, the abrupt yet small variations of EM-STR gains justify its enhanced adaptability, robustness, and smoother control activity under exogenous disturbances. This flexibility is attributed to the dynamic self-adjustment of all weighting-factors associated with the ARE. The enhanced adaptability of EM-STR comes at the cost of tuning a relatively large number of hyper-parameters (as compared to other adaptation mechanisms discussed here). However, the betterment in the performance is sufficient to ignore this drawback.

The experimental analysis validates the superior position-regulation accuracy and enhanced robustness of the EM-STR in almost every test-case. It manifests better adaptability under perturbed conditions as compared to other STC variants. It effectively removes the inherent shortcomings of other adaptation mechanisms, which enables it to flexibly steer the control trajectory. However, EM-STR does consume large control energy as compared to the other controllers in almost every test-case. The EP-STR shows the second-best time-domain performance after EM-STR.

The proposed hierarchical control procedure is highly scalable. Each controller variants exhibits a certain degree of resilience against the aforementioned disturbance scenarios. However, in future, the proposed control procedure can also be augmented with auxiliary neuro-fuzzy adaptive compensators, suggested in [72, 73], to effectively handle the hardware limits imposed on under-actuated systems; such as input and actuator dead-zones, limit cycles, and parametric uncertainties associated with the system’s actuated and un-actuated state-variables.

The constitution of the proposed hierarchical control procedure only requires the a priori identification of the systems linear state-space model and pre-calibrated weight-adjusting functions. Thus, apart from the self-stabilizing mechatronic platforms, the proposed control schemes can be easily extended to flexible-joint robotic manipulators and other classes on under-actuated systems as well [74].

## 6. Conclusion

This paper presents the comparative performance assessment of four state-error-driven hierarchical adaptive control strategies that enhance the disturbance-rejection capability of closed-loop under-actuated mechatronic systems. Each adaptation mechanism dynamically reconfigures the constituents of the Riccati equation in an innovative manner to self-tune the state-feedback gains of LQR. The proposed architecture delivers adaptive actions in real-time without explicitly relying on the estimation of state-dependent-coefficients in the system’s state-space model. This feature makes it highly scalable and computationally economical. The improvement in time-domain performance and robustness imparted by each self-tuning-regulators, discussed in this article, is analyzed under practical disturbance scenarios by conducting real-time hardware experiments on the QNET rotary pendulum system. The experimental outcomes validate the superior robustness and position-regulation accuracy of the EM-STR scheme in almost every test case. It is a resourceful scheme that utilizes the full state-error-feedback to self-adjust the state and control-input weighting-factors of the QPI online. The EP-STR delivers the second-best time-domain performance and maintains a reasonable control-input economy. Furthermore, EP-STR excels EM-STR under the influence of the step-disturbance. Its ability to self-mutate in real-time increases the controller’s degree-of-freedom which enhances the system’s response speed and damping against disturbances. In the future, the performance of the proposed control scheme can be further investigated by employing expert adaptive systems that are driven by soft computing techniques. The proposed reconfiguration schemes can also be enhanced by self-regulating the variances and exponents of the hyperbolic functions. The feasibility of the proposed controller(s) can also be analyzed by extending it to other mechatronic systems.

## Supporting information

S1 Dataset (XLSX)
